# Transcription-driven chromatin repression of Intragenic transcription start sites

**DOI:** 10.1371/journal.pgen.1007969

**Published:** 2019-02-01

**Authors:** Mathias Nielsen, Ryan Ard, Xueyuan Leng, Maxim Ivanov, Peter Kindgren, Vicent Pelechano, Sebastian Marquardt

**Affiliations:** 1 University of Copenhagen, Department of Plant and Environmental Sciences, Copenhagen Plant Science Centre, Frederiksberg, Denmark; 2 SciLifeLab, Department of Microbiology, Tumor and Cell Biology, Karolinska Institutet, Solna, Sweden; Swedish University of Agricultural Sciences (SLU), SWEDEN

## Abstract

Progression of RNA polymerase II (RNAPII) transcription relies on the appropriately positioned activities of elongation factors. The resulting profile of factors and chromatin signatures along transcription units provides a “positional information system” for transcribing RNAPII. Here, we investigate a chromatin-based mechanism that suppresses intragenic initiation of RNAPII transcription. We demonstrate that RNAPII transcription across gene promoters represses their function in plants. This repression is characterized by reduced promoter-specific molecular signatures and increased molecular signatures associated with RNAPII elongation. The conserved FACT histone chaperone complex is required for this repression mechanism. Genome-wide Transcription Start Site (TSS) mapping reveals thousands of discrete intragenic TSS positions in *fact* mutants, including downstream promoters that initiate alternative transcript isoforms. We find that histone H3 lysine 4 mono-methylation (H3K4me1), an *Arabidopsis* RNAPII elongation signature, is enriched at FACT-repressed intragenic TSSs. Our analyses suggest that FACT is required to repress intragenic TSSs at positions that are in part characterized by elevated H3K4me1 levels. In sum, conserved and plant-specific chromatin features correlate with the co-transcriptional repression of intragenic TSSs. Our insights into TSS repression by RNAPII transcription promise to inform the regulation of alternative transcript isoforms and the characterization of gene regulation through the act of pervasive transcription across eukaryotic genomes.

## Author summary

Genes represent DNA elements that are transcribed into mRNA. However, the position where transcription actually starts can be dynamically regulated to expand the diversity of RNA isoforms produced from a single gene. Functionally, alternative Transcription Start Sites (TSSs) may generate protein isoforms with differing N-terminal regions and distinct cellular functions. In plants, light signaling regulates protein isoforms largely through regulated TSS selection, emphasizing the biological significance of this mechanism. Despite the importance of alternative TSS selection, little is known about the underlying molecular mechanisms. Here, we characterize for the first time how transcription initiation from an upstream promoter represses alternative downstream promoter activity in plants. This repression mechanism is associated with chromatin changes that are required to maintain precise gene expression control. Specific chromatin signatures are established during transcription via dynamic interactions between the transcription machinery and associated factors. The conserved histone chaperone complex FACT is one such factor involved in regulating the chromatin environment along genes during transcription. We find that mutant plants with reduced FACT activity specifically initiate transcription from thousands of intragenic positions, thus expanding RNA isoform diversity. Overall, our study reveals conserved and plant-specific chromatin features associated with the co-transcriptional repression of downstream intragenic TSSs. These findings promise to help inform the molecular mechanism underlying environmentally-triggered TSS regulation in plants.

## Introduction

Plasticity at the beginning and end of transcription units multiplies the RNA species that can be generated from genomes. Many RNA species result from RNA Polymerase II (RNAPII) activity at genes and abundant non-coding genomic regions [1, 2]. Pervasive transcription results in overlapping transcripts, for example by initiating intragenic transcription leading to the production of alternative transcript isoforms [3]. Alternative Transcription Start Sites (TSSs) expand RNA isoform diversity, may result in functionally different RNA and proteins specific to disease, and allow for multiple transcriptional outputs from a single gene [4, 5]. However, the mechanisms of alternative TSS activation, repression, and regulation are poorly understood in higher eukaryotes.

Repression of a gene promoter by overlapping RNAPII transcription was originally described for two tandemly arranged human α-globin gene copies [6]. Read-through transcription from the upstream α-globin gene positions the downstream promoter in the middle of a transcription unit spanning both gene copies. Repression of a downstream promoter through the act of RNAPII transcription is referred to as Transcriptional Interference (TI) [7]. The core of this mechanism relies on the progression of RNAPII transcription through distinct stages [8]. Each stage is characterized by the co-transcriptional recruitment of factors involved in nascent RNA processing and chromatin modifications [9]. Dynamic phosphorylation of residues in the C-terminal YSPTSPS repeat region of the largest RNAPII subunit coordinates progression through the transcription cycle by recruiting stage-specific factors [10, 11]. Metagene analyses of stage-specific transcription factors and chromatin signatures in diverse organisms strikingly visualize many common changes associated with RNAPII progression from the beginning to the end of active transcription units [12–17]. For example, histone 3 lysine 4 methylation (H3K4me) states decrease from tri- (H3K4me3) to mono-methylation (H3K4me1) from the beginning to the end of yeast genes [18]. Such signatures provide a “positional information system” (POINS) for RNAPII to coordinate molecular events required for each stage of transcription [8].

An important functional outcome of co-transcriptional chromatin changes involves the suppression of transcriptional initiation from within transcription units (intragenic TSSs). Whereas TSSs in gene promoters are characterized by well-defined DNA *cis*-elements [19], the activity of intragenic TSSs is connected to the co-transcriptional chromatin environment [20]. Histone 3 lysine 36 methylation (H3K36me) is characteristic of RNAPII elongation in many organisms [21–23]. H3K36 tri-methylation (H3K36me3) prevents RNAPII transcription initiation from intragenic TSSs by mediating histone de-acetylation in yeast [24–26]. Chromatin-based repression of intragenic TSSs is also tightly linked to the activity of histone chaperones [27, 28]. The FACT (FAcilitates Chromatin Transcription) complex, consisting of SSRP1 and SPT16, contributes to this activity across taxa [29, 30]. *SPT16* was initially characterized as a *SPT* (*suppressor of Ty*) gene that is required for the suppression of gene promoters by read-through transcription initiating from adjacent upstream Ty or δ-element insertions [31, 32]. RNAPII read-through transcription of upstream genes due to inefficient termination can elicit suppression of downstream gene promoters by TI [7, 33, 34]. Transcripts overlapping gene promoters may also arise from RNAPII transcription of long non-coding RNAs (lncRNAs) and suppress initiation by FACT-dependent TI [35–38]. In mammals, a combination of FACT, H3K36me3, and gene-body DNA methylation suppress intragenic TSSs [39, 40]. Co-transcriptional chromatin signatures are largely common across species, yet their roles in the regulation of intragenic TSSs often await experimental validation.

Many factors characterizing POINS are active in plants [16, 41]. The *Arabidopsis* FACT complex is physically associated with multiple RNAPII elongation factors, chromatin modifiers, and elongation specific RNAPII isoforms [42, 43]. Reduced FACT activity results in developmental defects [44] that are linked to abnormal DNA methylation at heterochromatin [45] and imprinted loci [46]. However, the role of FACT in TSS selection in plants is unclear. Moreover, H3K36me3 localizes to promoter regions in *Arabidopsis*, whereas the di-methylated H3K36 variant (H3K36me2) associates to RNAPII elongation zones [47]. These data indicate that mechanisms in addition to those previously described in budding yeast may have evolved to repress intragenic TSSs in plants. Genome-wide TSS mapping in *Arabidopsis* suggests that a choice between alternative TSSs exists for most transcripts [48]. Protein isoform diversity control in response to light through regulated TSS choice underpins the biological significance of this mechanism [49]. Moreover, TSS choice may also regulate gene expression at the level of translation by the inclusion of an upstream open reading frame (uORF) [50]. Despite the functional significance of alternative TSS choice, little is known about the molecular mechanisms regulating this phenomenon in plants.

Here, we demonstrate the repressive effect of RNAPII elongation across gene promoters in *Arabidopsis*. We identify chromatin and RNAPII signatures associated with this form of gene regulation by “repressive transcription”. We uncover thousands of intragenic TSSs in *fact* mutants, revealing a role for FACT in preventing initiation of RNAPII transcription from within plant transcription units. Our analyses of chromatin signatures identify increased levels of the RNAPII elongation-associated H3K4me1 signal at intragenic sites that function as TSSs when FACT function is compromised. Thus, we resolve plant-specific molecular events repressing transcription initiation by the process of RNAPII elongation and highlight this mechanism for the first time in the context of a multicellular organism.

## Results

### Gene promoter repression by upstream RNAPII transcription in *Arabidopsis thaliana*

To investigate gene repression through the act of RNAPII transcription across promoter regions in higher organisms, we performed a literature screen of *Arabidopsis* T-DNA insertion mutants with loss-of-function phenotypes [51]. This specific type of T-DNA mutants must: 1.) be inserted upstream of gene promoter TSSs, 2.) show read-through transcription into downstream genes, and 3.) segregate as a recessive loss-of-function phenotype. Application of these criteria identified the *quasimodo1-1* (*qua1-1*) and *red fluorescence in darkness 1–1* (*rfd1-1*) mutants as candidate mutants for further analysis [52, 53].

**Fig 1:**
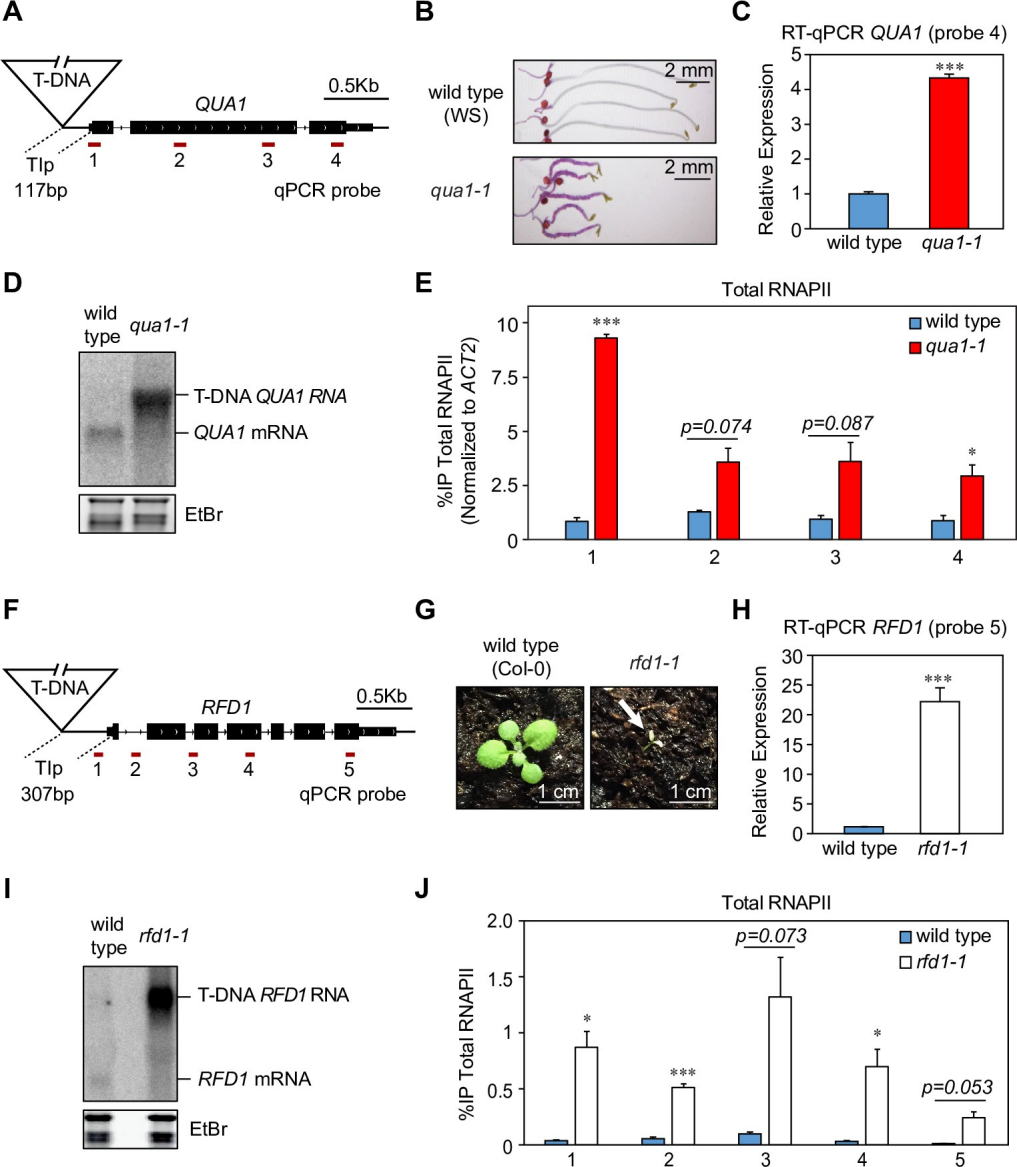
Upstream transcription from within T-DNA represses downstream gene expression. (A) Schematic representation of the *qua1-1* locus, including primer pair positions (probe 1–4) for qChIP and RT-qPCR. *TIp* denotes Transcriptionally Interfered promoter region remaining in the *qua1-1* mutant. (B) Ruthenium red staining of wild type (ecotype WS) and *qua1-1* hypocotyls. (C) Quantitative analysis of *QUA1* transcript levels in wild type and *qua1-1* by RT-qPCR using primer pair 4 (See Panel A, qPCR probe 4). (D) Analysis of *QUA1* transcripts in wild type and *qua1-1* by northern blotting. Ethidium bromide (EtBr) staining of ribosomal RNA is used as a control to assess relative equal loading and RNA quality. (E) qChIP for total RNAPII levels across *QUA1* in wild type and *qua1-1*. Note: For comparisons between wild type WS and *qua1-1*, qChIP values were normalized to reference gene *ACT2* in order to control for differential fixation conditions between samples (See methods for more details). (F) Schematic representation of the *rfd1-1* locus, including primer pair positions for qChIP and RT-qPCR (probes 1–5). *TIp* denotes Transcriptionally Interfered promoter region remaining in the *rfd1-1* mutant. (G) Photo-bleaching phenotype of *rfd1-1* seedlings grown in high light conditions (ecotype Col-0). (H) Quantitative analysis of *RFD1* transcript levels in wild type and *rfd1-1* by RT-qPCR using primer pair 5 (See Panel F, qPCR probe 5). (I) Analysis of *RFD1* transcripts in wild type and *rfd1-1* by northern blotting. EtBr staining of ribosomal RNA is used as a control to assess relative equal loading and RNA quality. (J) qChIP for total RNAPII %IP levels across *RFD1* in wild type and *rfd1-1*. Error bars represent standard error of the mean resulting from three independent replicates. For statistical tests, a single asterisk denotes p<0.05, two asterisks denote p<0.01, three asterisks denote p<0.001 between samples by Student’s t-test.

*QUA1* encodes a glycosyltransferase required for the biosynthesis of cell-adhesion promoting pectins [52]. The *qua1-1* T-DNA mutation is inserted 117 bp upstream of the annotated translational start site (Fig 1A; S1A Fig). The cell-adhesion defect in *qua1-1* results in dwarfed growth and ruthenium red staining of dark grown *qua1-1* hypocotyls (Fig 1B). We detect elevated *QUA1* expression in *qua1-1* compared to wild type by RT-qPCR (Fig 1C). Northern blotting reveals an abundant *T-DNA-QUA1* compound transcript in *qua1-1* instead of the *QUA1* mRNA (Fig 1D) [52]. The extended transcript detected in *qua1-1* corresponds to a predicted transcript initiating within the T-DNA and extending into the downstream *QUA1* gene (S1B Fig). Next, we performed quantitative chromatin immunoprecipitation (qChIP), which confirmed increased RNAPII levels across the *QUA1* gene in *qua1-1*, consistent with elevated levels of transcription initiating from within the T-DNA (Fig 1E). *RFD1* encodes RIBA1, the first enzyme in the plant riboflavin biosynthesis pathway [53]. The T-DNA insertion is located 307 bp upstream of the *RFD1* translational start site (Fig 1F; S1C Fig). Under standard light conditions, most soil-grown homozygous *rfd1-1* mutants die with white cotyledons (Fig 1G) [53]. However, we are now able to grow homozygous *rfd1-1* mutants to seed under reduced light conditions, enabling comparative analysis of the *RFD1* transcript pattern in wild type and homozygous *rfd1-1* mutants. Although RT-qPCR analysis shows about 20-times higher *RFD1* expression in *rfd1-1* compared to wild type (Fig 1H), northern blotting reveals an abundant *T-DNA-RFD1* compound transcript with increased transcript size in *rfd1-1* initiating from the upstream T-DNA insertion (Fig 1I, S1D Fig) [53]. Notably, the endogenous *RFD1* mRNA isoform is not detected in *rfd1-1*. Increased RNAPII levels across the *RFD1* gene in *rfd1-1* were also confirmed by qChIP (Fig 1J). Together, these complementary analyses in *qua1-1* and *rfd1-1* are consistent with the hypothesis that initiation from the downstream gene promoter is repressed through the act of RNAPII transcription.

**Fig 2:**
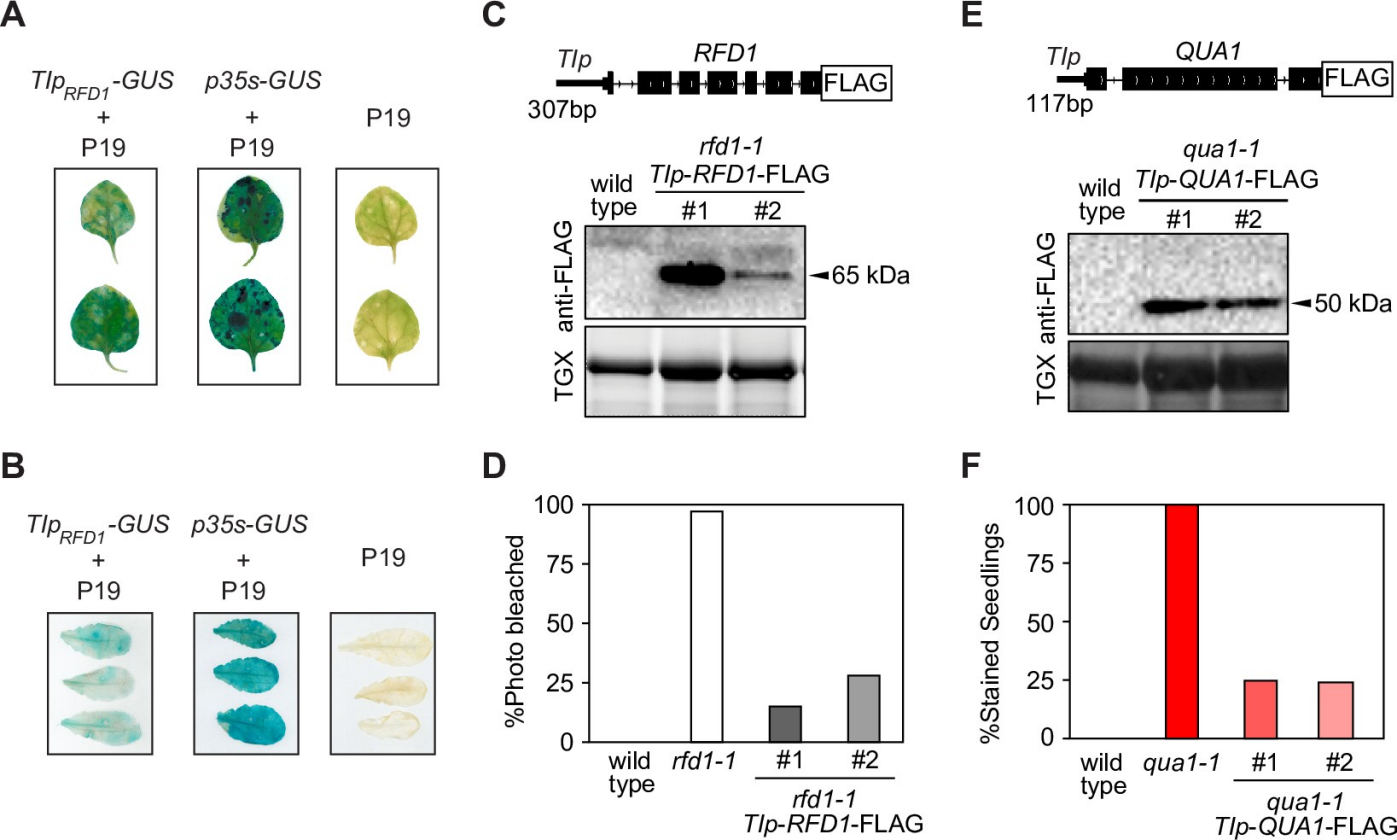
The act of upstream transcription represses a functional downstream promoter. (A) Transient expression of the *GUS* reporter gene under the control of *TIp*_*RFD1*_ in *N*. *benthamiana* leaves. *p35s-GUS* and *p19* (lacking GUS reporter gene) are used as positive and negative controls for GUS expression, respectively. (B) Transient expression of *GUS* reporter gene under the control of *TIp*_*RFD1*_ in leaves of the *A*. *thaliana efr* mutant. *p35s-GUS* and *p19* (lacking GUS reporter gene) are used as positive and negative controls for GUS expression, respectively. (C) Detection of RFD1-FLAG protein expressed from *TIp*_*RFD1*_*-RFD1-FLAG* by western blotting. For loading controls, total protein levels were detected using TGX stain-free protein gels. (D) Expression of RFD1-FLAG from *TIp*_*RFD1*_ complements *rfd1-1* photo-bleaching phenotype. Wild type (n = 84), *rfd1-1* (n = 143), lines #1 (n = 1196) and #2 (n = 943) segregating for the *TIp*_*RFD1*_*-RFD1-FLAG* complementation construct. (E) Detection of QUA1-FLAG protein from *TIp*_*QUA1*_*-QUA1-FLAG* by western blotting. For loading controls, total protein levels were detected using TGX stain-free protein gels. (F) Expression of QUA1-FLAG from *TIp*_*QUA1*_ complements ruthenium red staining *qua1-1* phenotype. Wild type (n = 97), *qua1-1* (n = 96), lines #1 (n = 267) and #2 (n = 254) segregating for the *TIp*_*QUA1*_*-QUA1-FLAG* complementation construct.

To test if the genomic region between the *rfd1-1* T-DNA insertion and the translational start site of *RFD1* can function as a promoter (designated as *TIp*_*RFD1*_, Fig 1F; S1C Fig), we assayed transient marker gene expression in *Nicotiana benthamiana* and *Arabidopsis thaliana* leaves. We detected expression of β-glucuronidase (GUS) (Fig 2A and 2B) and enhanced Yellow Fluorescent Protein (eYFP) (S2 Fig) driven by *TIp*_*RFD1*_ in transient expression assays. To test if *TIp* can drive gene expression in relevant tissues and at sufficiently high levels, we performed a molecular complementation of the read-through mutants with genomic constructs driven by their respective short *TIp*. We detect RFD1-FLAG protein expression in independent transformant lines by western blotting (Fig 2C). Importantly, RFD1 expression driven by *TIp*_*RFD1*_*-RFD1-FLAG* complements the *rfd1-1* phenotype (Fig 2D). Likewise, we detect QUA1-FLAG protein expression in independent *TIp*_*QUA1*_*-QUA1-FLAG* transformant lines by western blotting, and these lines complement the *qua1-1* phenotype (Fig 2E and 2F). Thus, *TIp* DNA regions provide necessary and sufficient promoter activity to drive functional *RFD1* or *QUA1* expression. Interfering RNAPII transcription across *TIp* is therefore a plausible mechanism to explain the repression of initiation despite transcriptional activity at these regions.

### Elevated RNAPII elongation signatures are found at promoters repressed through the act of upstream RNAPII transcription

Repressive RNAPII elongation across *TIp* in *qua1-1* and *rfd1-1* mutants may impact on molecular signatures associated with RNAPII elongation and initiation at *TIp*. To test this, we performed qChIP experiments to assay RNAPII initiation and elongation hallmarks. The elongating form of RNAPII (RNAPII-Ser2P) is enriched towards the 3’ end of the *QUA1* gene and depleted from the *QUA1* promoter in wild type *Arabidopsis* (S3A and S3B Fig). H3K36me3 is enriched towards the 5’ end of genes in *Arabidopsis*, while H3K36me2 corresponds to the elongation phase and accumulates towards the 3’ end [47]. We find the same pattern along the *QUA1* gene (S3C and S3D Fig). Histone modifications of active promoters such as histone H3 acetylation (H3ac) and H3K4me3 are enriched towards the *QUA1* promoter (S3E and S3F Fig) [47, 54, 55]. Thus, RNAPII initiation and elongation can be distinguished by our qChIP analyses.

**Fig 3:**
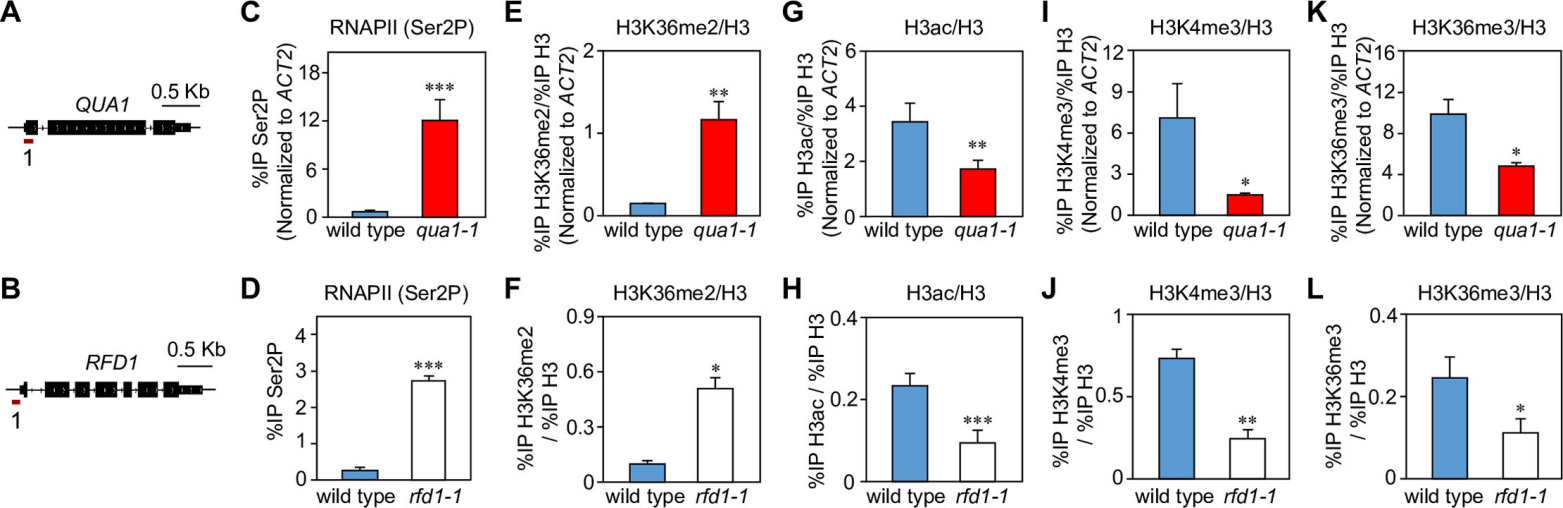
Promoters repressed by upstream transcription adopt RNAPII elongation signatures. (A) Schematic representation of the *QUA1* gene, including promoter-proximal primer pair position for qChIP. (B) Schematic representation of the *RFD1* gene, including promoter-proximal primer pair position for qChIP. (C-D) qChIP in mutants and their respective wild type ecotypes using promoter-proximal primer pairs for the elongating form of RNAPII (Ser2P). qChIP in mutants and their respective wild type ecotypes using promoter-proximal primer pairs for histone 3 (H3) modifications are shown (E-L). Data are normalized to H3 and show: (E, F) H3K36me2/H3, (G, H) H3 pan-acetylation (H3ac/H3), (I, J) H3K4me3/H3, and (K, L) H3K36me3/H3. Note: For comparisons between wild type (WS) and *qua1-1*, qChIP values were normalized to reference gene *ACT2* (See methods for more details). Error bars represent standard error of the mean resulting from at least three independent replicates. For statistical tests, a single asterisk denotes p<0.05, two asterisks denote p<0.01, three asterisks denote p<0.001 between samples by Student’s t-test.

We profiled *qua1-1* and *rfd1-1* mutants by qChIP to determine the impact of upstream RNAPII transcription across *TIp*_*QUA1*_ and *TIp*_*RFD1*_ (Fig 3A and 3B). Compared to their respective wild type ecotype, significantly higher levels of RNAPII-Ser2P were present at the position of promoter-proximal primer pairs in *qua1-1* and *rfd1-1* (Fig 3C and 3D). These results support increased RNAPII elongation across the downstream promoter. Since bulk histone density remains largely unchanged across *QUA1* and *RFD1* in their respective mutants (S3G–S3I Fig), we tested the presence of the *Arabidopsis* RNAPII elongation-specific chromatin signature H3K36me2. The mutants displayed increased H3K36me2 levels at *TIp*_*QUA1*_ and *TIp*_*RFD1*_ (Fig 3E and 3F). The increase of RNAPII elongation signatures at these promoters during repression indicates that these regions may now identify as zones of RNAPII elongation, rather than promoters. Consistent with this hypothesis, histone modifications associated with active promoters (H3ac, H3K4me3, H3K36me3) were significantly depleted at *TIp*_*QUA1*_ and *TIp*_*RFD1*_ in the mutants (Fig 3G–3L). Collectively, these results demonstrate that upstream RNAPII transcription shifts the POINS to specify downstream promoters as intragenic regions. Our data suggest that promoter repression in these mutants could be driven by transcription-mediated chromatin state changes.

### *Arabidopsis* FACT is required for gene repression through the act of upstream RNAPII transcription

**Fig 4:**
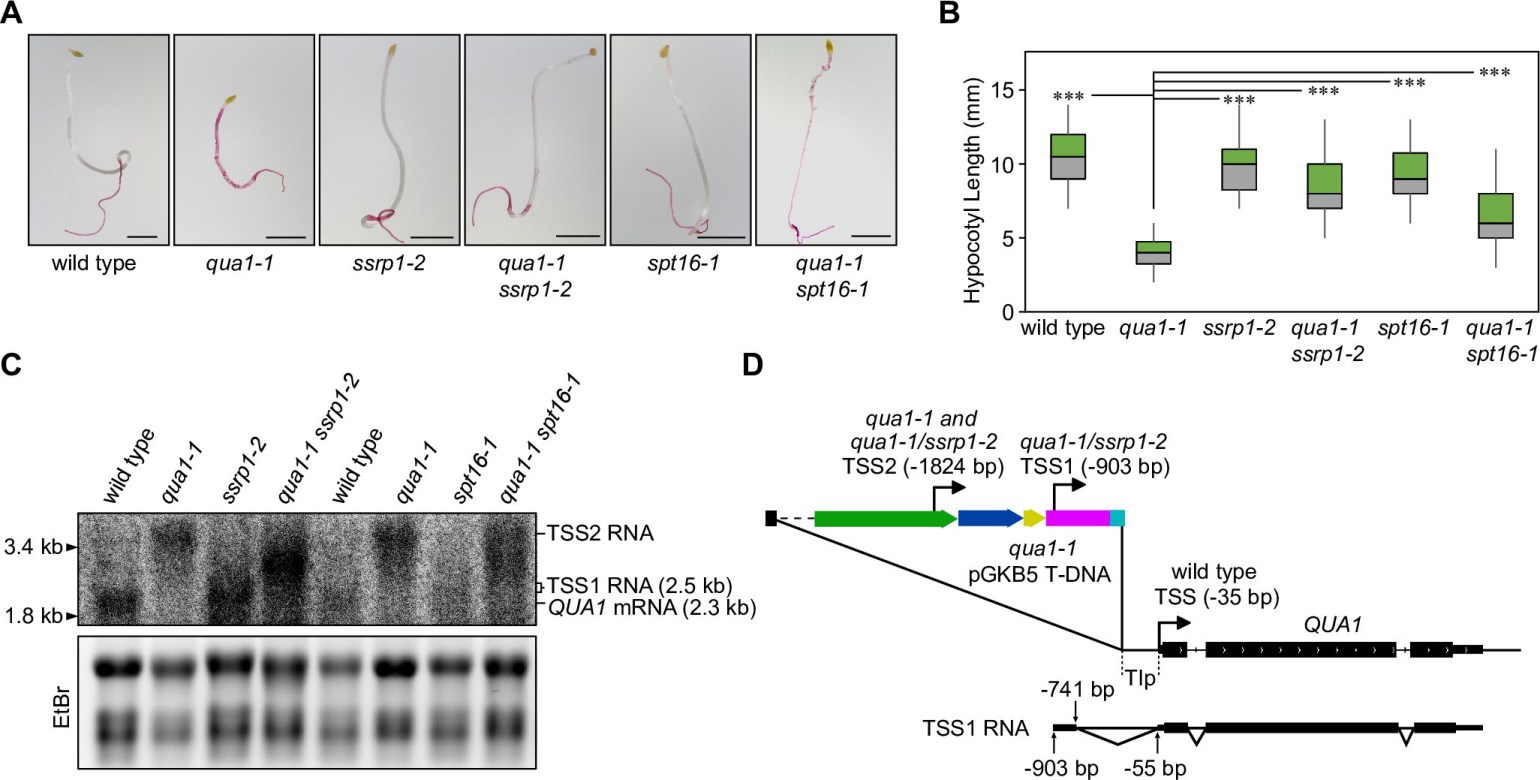
The *Arabidopsis* FACT complex is required for downstream gene repression through the act of RNAPII transcription. (A) Ruthenium red staining of wild type (WS), *qua1-1*, *spt16-1*, *ssrp1-2*, and double mutants *qua1-1/spt16-1* and *qua1-1/ssrp1-2*. All scale bars represent 2 mm. (B) Quantification of hypocotyl length (mm) for 7 days, dark-grown wild type (WS), (n = 30), *qua1-1* (n = 30), *spt16-1* (n = 30), *ssrp1-2* (n = 30), and double mutants *qua1-1/spt16-1* (n = 30) and *qua1-1/ssrp1-2* (n = 30). Three asterisks denote p<0.001 between *qua1-1* and all other samples by Student’s t-test. (C) Analysis of *QUA1* transcripts in wild type (WS), *qua1-1*, *ssrp1-2*, and the *qua1-1/ssrp1-2* double mutant as well as in *spt16-1* and the *qua1-1/spt16-1* double mutant by northern blotting. EtBr staining of ribosomal RNA is used as a control to assess relative equal loading and RNA quality. (D) Schematic representation of the *qua1-1* locus with T-DNA insertion (p35s: green, bialaphos resistance gene: blue, terminator: yellow, left border: magenta, polylinker: cyan) including the positions of TSSs mapped by 5’RACE in wild type (WS), *qua1-1*, and the *qua1-1/ssrp1-2* double mutant. TSS (-35 bp) represents the canonical transcription start site of *QUA1* in wild type. TSS2 (-1824 bp) represents the upstream TSS originating from p35s in the T-DNA insertion, shared by *qua1-1* and *qua1-1/ssrp1-2* double mutant. TSS1 (-903 bp) represents the new TSS in *qua1-1/ssrp1-2*. TSS1 results in a novel spliced *QUA1* mRNA transcript with a short 5’UTR extension (182 nt) that corresponds to a functional *QUA1* isoform.

Our analyses support that gene promoters can be repressed by interfering RNAPII elongation in *Arabidopsis*. We hypothesized that factors associated with RNAPII elongation, such as the FACT complex, may be required for repression. To test the role of FACT in promoter repression by read-through transcription in *Arabidopsis*, we combined the previously described knock-down alleles of *spt16-1* and *ssrp1-2* mutants with *qua1-1* [44]. Ruthenium red staining comparing single and double mutants revealed patches of unstained hypocotyls in *spt16-1 qua1-1* compared to *qua1-1* (Fig 4A). Importantly, *spt16-1 qua1-1* alleviated the dwarf hypocotyl phenotype observed in *qua1-1* (Fig 4B). These results indicate tightened cell-adhesion and partial suppression of the *qua1-1* phenotype. The rescue effect was even more pronounced in *ssrp1-2 qua1-1* compared to *spt16-1 qua1-1* (Fig 4A and 4B). This can be explained by stronger knock-down of protein levels in *ssrp1-2* compared to *spt16-1* [44]. To test if FACT was required for read-through repression of *RFD1* in *rfd1-1*, we crossed *spt16-1* with *rfd1-1*. In our experimental conditions, about 20% of the progeny of heterozygous *rfd1-1/RFD1* seeds segregate for the photo-bleaching phenotype (S4A Fig). The progeny of seed segregating in addition for SPT16/*spt16-1* reduced the photo-bleaching phenotype by about 25%, consistent with suppression of photo-bleaching in the *rfd1-1 spt16-1* mutant (S4A Fig). Collectively, these results support the conclusion that FACT is genetically required for interfering RNAPII elongation at the *qua1-1* and *rfd1-1* alleles.

To test the roles of additional RNAPII elongation factors in repression, we assayed genetic interactions between *qua1-1* and mutations in the *Arabidopsis* PAF-I (Polymerase-Associated Factor I) subunit *VIP6* [56] and the Elongator subunit *ELO3* [57]. To examine the role of H3K36me2, we tested the interaction between *qua1-1* and a mutation in the H3K36 methyltransferase *SDG8/ASHH2* [58, 59]. Interestingly, unlike *spt16-1*, we find no evidence for suppression of *qua1-1* in these mutants (S4B Fig). Genetic linkage between *QUA1* and *SSRP1* precluded the inclusion of *ssrp1-2* in this assay (S4C Fig). All in all, these data argue for a key contribution of FACT during RNAPII elongation to trigger the *qua1-1* phenotype.

If phenotypic suppression of *qua1-1* through *fact* mutants was mechanistically linked to gene repression through the act of upstream interfering transcription, we would predict transcriptional changes. To examine the pattern of *QUA1* transcripts, we performed northern blotting in single and double mutants. While the transcript pattern in *spt16-1* and *ssrp1-2* is not clearly distinguishable from wild type controls, we observe new transcript patterns in *spt16-1 qua1-1* and *ssrp1-2 qua1-1* double mutants compared to *qua1-1* (Fig 4C). Importantly, variants of the high-molecular weight interfering transcripts remain detectable in *fact qua1-1* double mutants, suggesting that upstream interfering transcription can still be initiated. The interfering transcript in *fact qua1-1* double mutants appears to have a more broad size distribution than in *qua1-1*, which is revealed most clearly by a reduced size of the main interfering transcript isoform in *ssrp1-2 qua1-1*. While we find no evidence for the *QUA1* mRNA in *qua1-1*, we detect hybridization signal in *fact qua1-1* double mutants overlapping the expected size of the QUA1 mRNA transcript. These data suggested one or more 5’-truncated transcripts initiating from cryptic TSSs in *fact qua1-1* double mutants that could restore functional QUA1 expression.

To resolve such transcripts, we performed 5’ Rapid Amplification of cDNA Ends (5’RACE) in the *ssrp1-2 qua1-1* double mutant compared to *qua1-1*. Even though there appear to be differences in the main interfering transcript size in *qua1-1* compared to *ssrp1-2 qua1-1* (Fig 4C), our 5’RACE identifies a common TSS (TSS2) in these genotypes (Fig 4D). Importantly, we identified a novel TSS (TSS1) in *ssrp1-2 qua1-1* (Fig 4D). While TSS1 does not match the exact wild type *QUA1* mRNA in *ssrp1-2 qua1-1*, usage of TSS1 results in a short (182 nt) 5’-extension of the *QUA1* mRNA. It remains possible that the wild type *QUA1* TSS may also be used in *ssrp1-2 qua1-1* but was not captured by our 5’RACE experiments. Phenotypic suppression indicates that functional *QUA1* mRNAs are produced from cryptic TSSs, such as TSS1, that are accessible in *fact* mutants despite interfering transcription across the *QUA1* promoter region. Overall, our results support the conclusion that the activity of the FACT complex as part of RNAPII elongation suppresses TSSs inside of transcription units.

### *Arabidopsis* FACT restricts the activity of intragenic transcription start sites

**Fig 5:**
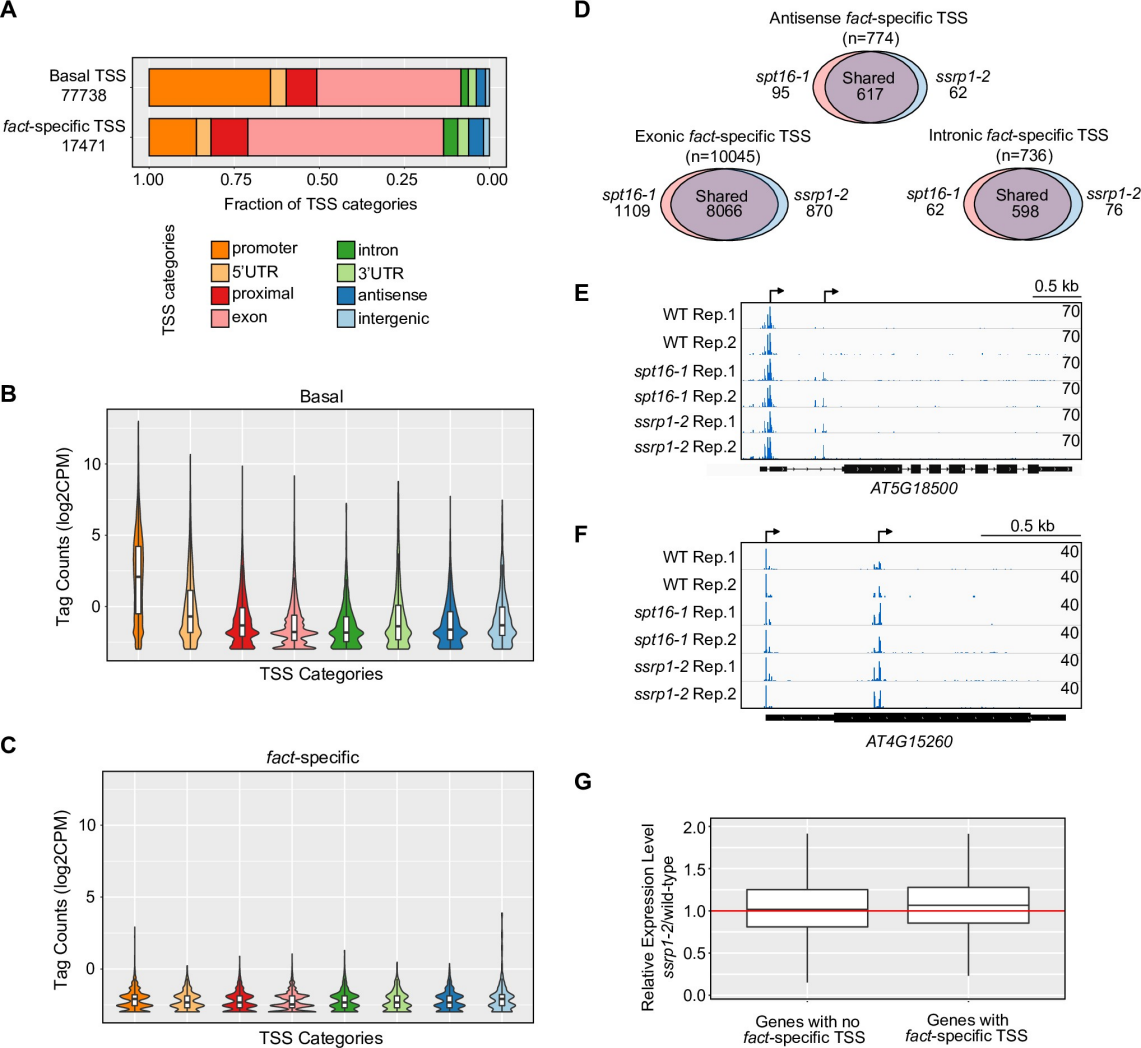
FACT represses intragenic TSSs across the *Arabidopsis* genome. (A) Genomic annotations of the basal set (upper, n = 77738) and the *fact*-specific set of TSSs (lower, n = 17471). (B, C) Distribution of log2-transformed expression values within each annotation category of the basal set (B) and the *fact*-specific set of TSS (C). (D) Venn diagrams visualizing the overlap of exonic, intronic and antisense *fact*-specific TSSs in *spt16-1* and *ssrp1-2*. (E) Genome browser screenshot of TSS-seq data showing an intronic *fact*-specific intragenic TSS observed in the *AT5G18500* gene. *AT5G18500* gene architecture is given below, the *fact*-specific TSSs and the promoter TSSs are indicated by black arrows (top) (F) Genome browser screenshot of TSS-seq data showing a *fact*-specific TSS for the *AT4G15260* gene. *AT4G15260* gene architecture is given below, the intragenic TSSs with increased TSS-seq signal in *fact* mutants and the promoter TSSs are indicated by black arrows (top) (G) Quantification of gene promoter expression (*ssrp1-2*/wild-type) between genes with and without *fact*-specific TSSs. Analyses are based on TSS-seq data.

To test if FACT suppresses endogenous intragenic TSSs, we measured *Arabidopsis* TSSs by 5’-CAP-sequencing (TSS-seq) [60]. We obtained on average 47 million raw reads for two biological repeats of wild type, *spt16-1*, and *ssrp1-2* (S1 Table). We identified 96232 TSS clusters and annotated them by genomic location. Many TSS clusters (n = 30487, or 31.7%) mapped to annotated gene promoters (Fig 5A; S5A Fig). The number of sequencing reads supporting TSS clusters showed a high degree of correlation between biological repeats (S5B Fig). We examined the overlap of our TSS clusters with TSSs identified by CAGE (Cap Analysis Gene Expression) [48]. 76.7% of TSS clusters in annotated gene promoters overlap with at least one previously reported CAGE peak (S5C Fig and S2 Table), indicating very good overlap across techniques and samples. Alternative mRNA isoforms of *AT4G08390* are differentially targeted to mitochondria or chloroplast [61]. Our data resolve TSSs corresponding to these isoforms (S5D Fig). Interestingly, our TSS-seq data reveals 17.4-fold more TSSs in exons (n = 43414, or 45.1%) than in introns (n = 2460, or 2.5%) (Fig 5A and S3 Table). The *Arabidopsis* genome contains 2.6-fold more exonic bases (51.6 Mb) than intronic bases (19.7 Mb), offering a partial explanation for the biased location of intragenic TSSs in exons. In conclusion, these data illustrate high reproducibility of our TSS-seq methodology, and its abilities to validate TSSs as well as to reveal novel TSSs.

To test the role of FACT in regulating TSSs in *Arabidopsis*, we divided the TSSs into three groups (S4 Table): i) basal TSSs detected in both wild type and *fact* mutants (n = 77738, or 80.8%); ii) wild-type specific TSSs (n = 1023, or 1.06%); and iii) TSSs specifically detected in *fact* mutants (i.e. *fact*-specific TSSs; n = 17471, or 18.1%, S3 Table). The 17-fold increase of *fact*-specific TSSs over the wild-type specific TSSs suggests that the FACT complex largely represses TSSs. We frequently find *fact*-specific TSSs in intragenic locations (Fig 5A). However, TSSs induced in *fact* mutants have a lower TSS-seq count compared to the basal TSS set indicating lower expression of transcripts derived by *fact*-specific TSSs (Fig 5B and 5C). The large majority of *fact*-specific TSSs (9281 out of 11555, or 80.3%) were detected in both *fact* mutants (Fig 5D). As much as 83.1% of *fact*-specific exonic TSSs do not overlap with a TSS identified by CAGE (S5C Fig and S2 Table). The *AT5G18500* gene illustrates the induction of an intronic TSS in *fact* mutants (Fig 5E). The *AT4G15260* UDP-glycosyltransferase gene reveals preferential usage of a downstream intragenic TSS in *fact* mutants that is normally regulated in response to light signaling (Fig 5F) [49], suggesting that the promoter for the shorter transcript isoform is suppressed in a FACT-dependent manner from upstream RNAPII transcription. We next quantified TSS-seq peaks at canonical promoters for genes with or without *fact*-specific TSSs and compared their expression in wild type and *ssrp1-2*. These analyses reveal that expression of the isoforms initiating at the canonical promoter TSSs for genes with *fact*-specific TSSs show no significant genome-wide decrease in *ssrp1-2* (Fig 5G). These data indicate that initiation from intragenic *fact*-specific TSSs does not necessarily result from reduced transcription initiating from upstream promoters, arguing against a promoter competition model. Overall, our TSS-seq data reveal thousands of intragenic regions that can function as TSSs depending on FACT activity. These results support a role of FACT as part of POINS in *Arabidopsis*, with a key function in suppressing intragenic TSSs.

### Chromatin-state analyses of intragenic regions that function as TSSs in *fact* mutants

**Fig 6:**
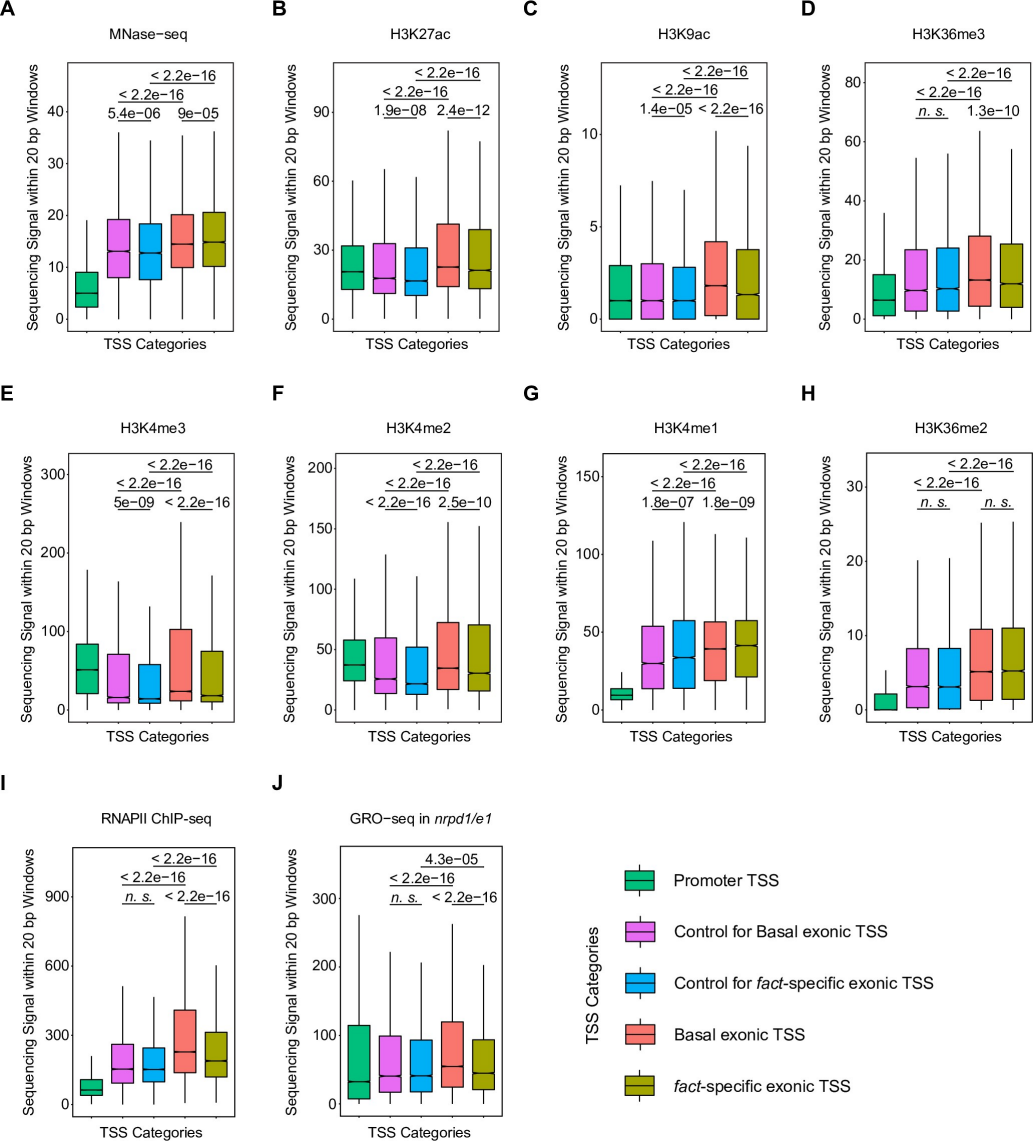
H3K4me1 is enriched at FACT-repressed TSS positions. Boxplots show the distribution of median ChIP-seq, GRO-seq and MNase-seq signals within 20 bp windows centered at the following positions: i) Promoter TSS (green); ii) Control exonic positions in genes with basal exonic TSS (purple); iii) Control exonic position in genes with *fact*-specific TSS (blue); iv) Basal exonic TSS (salmon); v) *fact*-specific exonic TSS (olive). The notch denotes the median value, hinges denote quartiles and whiskers show the spread of non-outlier values (found within 1.5*IQR from the respective quartile). The p-values were calculated by Wilcoxon test. The following datasets were included: (A) MNase-seq; (B) H3K27ac; (C, D) H3K9ac and H3K36me3; (E, F, G) H3K4me3, H3K4me2 and H3K4me1; (H) H3K36me2; (I) RNAPII ChIP-seq; (J) GRO-seq.

Common DNA sequences or chromatin signatures may predispose intragenic regions to function as *fact*-specific TSSs. We tested differential DNA-motif enrichment in exonic *fact*-specific TSSs compared to basal exonic TSSs. However, we detect no differentially enriched sequence motif or position bias within exons (S5E Fig). To test if exonic TSSs may be characterized by promoter-like chromatin architecture, we re-analyzed available *Arabidopsis* ChIP-seq data of chromatin signatures in wild type [62–66]. We compared chromatin signatures centered on five sets of genomic locations: *fact*-specific exonic TSS positions, exonic control regions without TSSs in the same set of genes that have *fact*-specific exonic TSSs, basal exonic TSSs, exonic control regions without TSSs in the same set of genes that have basal exonic TSSs, and TSSs at gene promoters. Box plots capturing the median sequencing signal in 20 bp intervals around the positions are given to present data variability and associated statistical tests between the five genomic sets (Fig 6, S7 Fig). Metagene plots of the mean sequencing signal in a 400 bp interval centered on the positions are given with standard errors to visualize the dynamics of the chromatin signatures around the positions (S6 and S7 Figs). *Arabidopsis* promoter-chromatin signatures clearly distinguish TSSs identified in gene bodies from TSSs at gene promoters (Fig 6), which is well-illustrated through the shape of accumulated signal in the metagene plots (S6 Fig). Promoter TSSs show low nucleosome signal assayed by MNase-seq compared to intragenic TSSs and control regions (Fig 6A, S6A Fig). *fact*-specific exonic TSSs show the highest MNase-seq signal compared to basal exonic TSSs and control regions. These data argue against promoter-like, low nucleosome density at *fact*-specific exonic TSSs in the repressed state. Moreover, the set of basal exonic TSSs is often enriched for promoter chromatin-signatures compared to *fact*-specific TSSs (Fig 6B–6F, S6B–S6F Fig). These data argue against the idea that exonic regions we identify as *fact*-specific TSSs show the chromatin architecture of promoter TSSs in wild type.

Of all ChIP-seq experiments assaying histone modifications that we analyzed, histone 3 lysine 4 mono-methylation (H3K4me1), associated with RNAPII elongation, represents the only post-translational histone modification that is enriched at *fact*-specific TSSs compared to control regions and basal exonic TSSs (Fig 6G p = 1.8e^-09^, S6G Fig, S7 Fig). The levels of H3K36me2, an alternative *Arabidopsis* RNAPII elongation signature, between basal TSSs and *fact*-specific TSSs are indistinguishable (Fig 6H). These data argue for a differential effect of *Arabidopsis* elongation-specific chromatin signatures, consistent with distinct contributions of the FACT complex among RNAPII elongation factors suggested by the genetics (S4 Fig). To test if the detected increase of H3K4me1 at *fact*-specific TSSs could be explained by a bias in the particular ChIP-seq data [63], we analyzed ChIP-seq data generated by an independent study that also assayed all three methylation states of H3K4 [67]. Consistently, the data for H3K4 di-and tri-methylation resulted in overall similar profiles (Fig 6E and 6F, S6E and S6F Fig, S7A and S7B Fig). Importantly, the increase of H3K4 mono-methylation at exonic *fact*-specific TSSs could be confirmed (S7C Fig p = 2.4e^-08^). The combination of H3K4me1 and H3K27ac chromatin signatures characterizes enhancers in many systems. However, even though *fact*-specific TSSs appear enriched in H3K4me1, these sites are reduced in H3K27ac compared to basal exonic TSSs (Fig 6B, p = 2.4e^-12^). Our analyses offer no evidence to support the idea that locations of *fact*-specific TSSs may represent intragenic enhancers. In summary, these analyses suggest that exonic *fact*-specific TSSs carry chromatin signatures of RNAPII elongation that are enriched for H3K4me1.

While the FACT complex directly interacts with residues in H3/H4 [68–70], it interacts more strongly with H2A/H2B dimers and is considered a H2A/H2B chaperone in many organisms, including *Arabidopsis* [71, 72]. To test if chromatin signatures based on H2A/H2B may participate in predisposing exonic sites as TSSs in *fact* mutants, we analyzed wild-type ChIP-seq data for H2A, ubiquitinylation at H2A lysine 121 (H2AUb), H2B, mono-ubiquitinylation of H2B lysine 120 (H2BUb) and the H2A variant H2A.Z [73–76]. H2A.Z and H2Aub match the profiles of chromatin signatures of promoter TSSs, whereas we detect the strongest H2A signal in exons (S7D–S7F Fig). However, H2A.Z levels at *fact*-specific TSSs are indistinguishable from those at basal exonic TSSs, arguing against a role of H2A.Z in specifying *fact*-specific TSSs. We note that basal exonic TSSs are enriched for H2AUb compared to *fact*-specific TSSs and control regions, consistent with elevated H3-based promoter TSSs chromatin signatures. The profile of H2B ChIP-seq data matches those of promoter TSSs-associated chromatin signatures, whereas H2BUb is enriched in exons, consistent with previously suggested roles in RNAPII elongation (S7G and S7H Fig). Quantification of ChIP-seq signal identified no statistically significant changes between *fact*-specific exonic TSSs and basal exonic TSSs. Perhaps surprisingly, given the preferential activity of FACT as an H2A/H2B chaperone, our analyses found no evidence for H2A or H2B-based chromatin signatures distinguishing *fact*-specific TSSs that may mark these locations in concert with H3K4me1.

To test if *fact*-specific intragenic TSSs present in exons enriched for H3K4me1 may be a consequence of high RNAPII transcription, we assessed RNAPII occupancy using RNAPII ChIP-seq data [64]. To assay transcriptionally active populations of RNAPII we analyzed Global Run-On sequencing data (GRO-seq) [77]. We used GRO-seq data generated in *nrpd1/nrpe1* double mutants to ensure the GRO-seq signal is specific to RNAPII, as previously described [77]. Interestingly, exonic regions identified as TSSs accumulate more RNAPII compared to exonic control regions in the same gene sets (Fig 6I, S6I Fig), and this fraction of RNAPII is transcriptionally active (Fig 6J, S6J Fig). Basal exonic TSSs correspond to more highly transcribed regions than *fact*-specific exonic TSSs (Fig 6I and 6J), arguing against the idea that *fact*-specific TSSs represent regions with particularly high RNAPII activity. In conclusion, our chromatin-state analyses focused on exonic TSSs suppressed by FACT are consistent with a co-transcriptional mechanism that may be linked to the H3K4me1 chromatin signature.

### FACT-mediated repression of intragenic TSSs is associated with H3K4 methylation state dynamics

**Fig 7:**
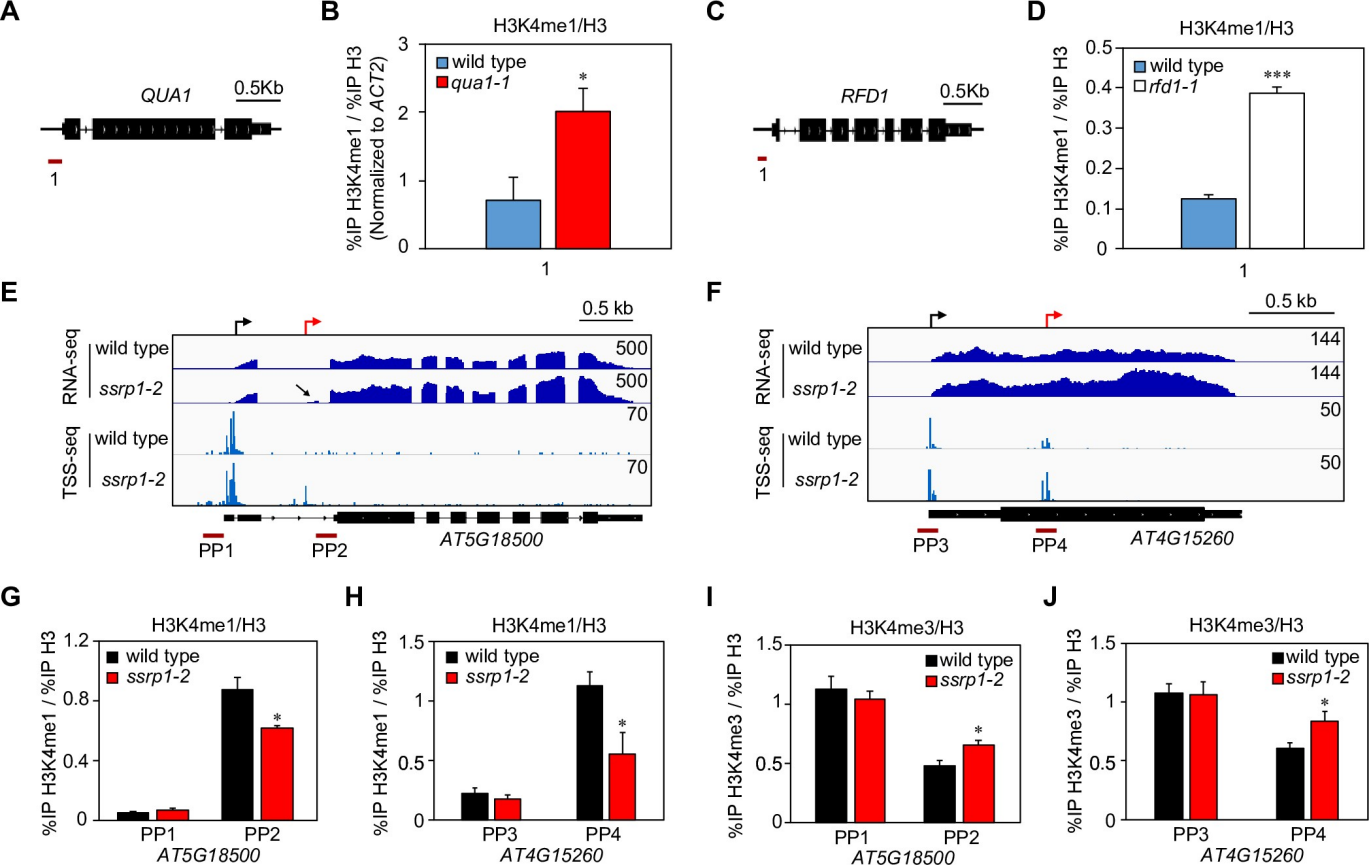
Activation of intragenic TSSs in *fact* mutant accompanies a shift from H3K4me1 to H3K4me3. (A) Schematic representation of the *QUA1* gene, including primer pair positions for qChIP. (B) qChIP in wild type (WS) and *qua1-1* using the *QUA1* promoter-proximal primer pair 1 (See panel A). Note: For comparisons between wild type WS and *qua1-1*, qChIP values were normalized to reference gene *ACT2* (See methods for more details). (C) Schematic representation of the *RFD1* gene, including primer pair positions for qChIP. (D) qChIP for H3K4me1 in wild type (Col-0) and *rfd1-1* using *RFD1* promoter-proximal primer pair 1 (See panel C). Screenshot of RNA-seq and TSS-seq data from wild type and *ssrp1-2* depicting novel intragenic transcripts emerging from *fact*-specific TSSs (red arrows) at the (E) *AT5G18500* and (F) *AT4G15260* genes. qChIP for H3K4me1 levels at (G) the canonical *AT5G18500* promoter (PP1) and *fact*-specific TSS (PP2) as well as at (H) the canonical *AT4G15260* promoter (PP3) and *fact*-specific TSS (PP4) in wild type and *ssrp1-2*. qChIP for H3K4me3 levels at (I) the canonical *AT5G18500* promoter (PP1) and *fact*-specific TSS (PP2) as well as at (J) the canonical *AT4G15260* promoter (PP3) and *fact*-specific TSS (PP4) in wild type and *ssrp1-2*. Error bars represent standard error of means resulting from at least three independent replicates. For statistical tests, a single asterisk denotes p<0.05 between samples by Student’s t-test.

Our above analyses of available ChIP-seq datasets suggest that at least part of the specification mechanism that distinguishes exonic regions to function as TSSs in *fact* mutants from basal exonic TSSs may involve relatively high starting levels of H3K4me1. As the chromatin signatures of the *QUA1* and *RFD1* promoter region in their respective *qua1-1* and *rfd1-1* mutants were not assayed by the wild type ChIP-seq data, we tested if these promoter regions also showed high H3K4me1 in *qua1-1* and *rfd1-1* read-through mutants. Indeed, we detected increased H3K4me1 in the mutants compared to their respective wild type controls (Fig 7A–7D). These results are consistent with FACT-dependent repression of TSSs around the promoter regions of the *RFD1 and QUA1* genes when these promoter regions acquire RNAPII elongation signatures such as H3K4me1 by read-through transcription in *qua1-1* and *rfd1-1* mutants.

To test if the repression of gene promoter TSSs by RNAPII elongation shares molecular signatures of TSS repression within gene bodies, we performed targeted qChIP analyses at selected *fact*-specific intragenic TSSs comparing wild type and the *ssrp1-2* mutant. To identify endogenous genes for qChIP analysis, we selected strongly induced intragenic *fact*-specific TSSs. We next performed RNA-seq in wild type and *ssrp1-2* to refine our selection based on a visual increase of RNA-seq reads in exons downstream of *fact*-specific TSSs in the *ssrp1-2* sample. The increased RNA-seq signal downstream of *fact*-specific TSSs implies that these TSSs generate *bona fide* alternative transcripts. We selected four genes with intragenic *fact*-specific TSSs (*AT5G18500*, *AT4G15260*, *AT3G56210*, and *AT5G51200*) and two control loci for basal exonic TSSs (*AT5G13630* and *AT1G06680*) (Fig 7E and 7F and S8A–S8D Fig). We measured H3K4me1 and H3K4me3 levels by qChIP in wild type and *ssrp1-2* at promoter TSSs and intragenic TSSs for the four genes with intragenic *fact*-specific TSSs and at the two basal exonic TSSs. We present these data normalized to H3 signal at these positions to account for potential changes in H3 levels. Importantly, triggering intragenic TSSs in *ssrp1-2* corresponded to a significant decrease in H3K4me1 at the four *fact*-specific TSSs (Fig 7G and 7H, S8E and S8F Fig), whereas we could detect no difference at the basal exonic TSSs (S8G and S8H Fig). Conversely, H3K4me3 levels increase at all four *fact*-specific TSSs in *ssrp1-2* mutants (Fig 7I and 7J, S8I and S8J Fig), whereas we could not detect any change at the basal exonic TSSs (S8K and S8L Fig). We note that the levels of these marks at the corresponding gene promoter TSSs are not significantly changed (Fig 7G and 7J, S8 Fig), offering chromatin-based support that the overall expression of the gene isoforms starting at the promoter TSSs are largely unaffected by this mechanism. These findings are consistent with our genome-wide TSS-seq analyses of promoter TSS strength for genes with and without *fact*-specific TSSs (Fig 5G). In conclusion, our qChIP analyses of H3K4 mono- and tri-methylation states suggest dynamic changes when FACT activity is compromised: *fact*-specific intragenic TSSs acquire H3K4me3 chromatin signatures of active promoters that are correlated with a reduction of H3K4me1.

Given the function of FACT as a histone chaperone, it seems plausible that a reduction in nucleosome density in *fact* mutants may facilitate the establishment of *fact*-specific TSSs. While we detected a trend of reduced bulk H3 levels at *fact*-specific TSSs in the *ssrp1-2* mutant, these changes were statistically significant at only three of six intragenic loci tested (S9 Fig). These data suggest that reduced nucleosome density in *ssrp1-2* may aid the formation of *fact*-specific exonic TSSs, yet does not offer a satisfactory explanation for this phenomenon. We examined possible changes in the presence of other histone modifications by qChIP: two active promoter marks (H3K36me3 and H3K27ac) and an elongation mark (H3K36me2). We observed a general trend, although not always statistically significant, towards increased H3K36me3 and H3K27ac at *fact*-specific TSSs in *ssrp1-2*, while H3K36me2 was generally reduced (S10–S12 Figs). Importantly, we did not detect significant changes in the levels of any of the histone modifications tested at the control basal TSS positions in *ssrp1-2* (S8–S12 Figs). Collectively, our qChIP analyses suggest that FACT represses intragenic TSSs co-transcriptionally by regulating chromatin changes that favor a balance of relatively high intragenic H3K4me1 levels and low levels of chromatin signatures found at promoter TSSs, such as H3K4me3.

**Fig 8:**
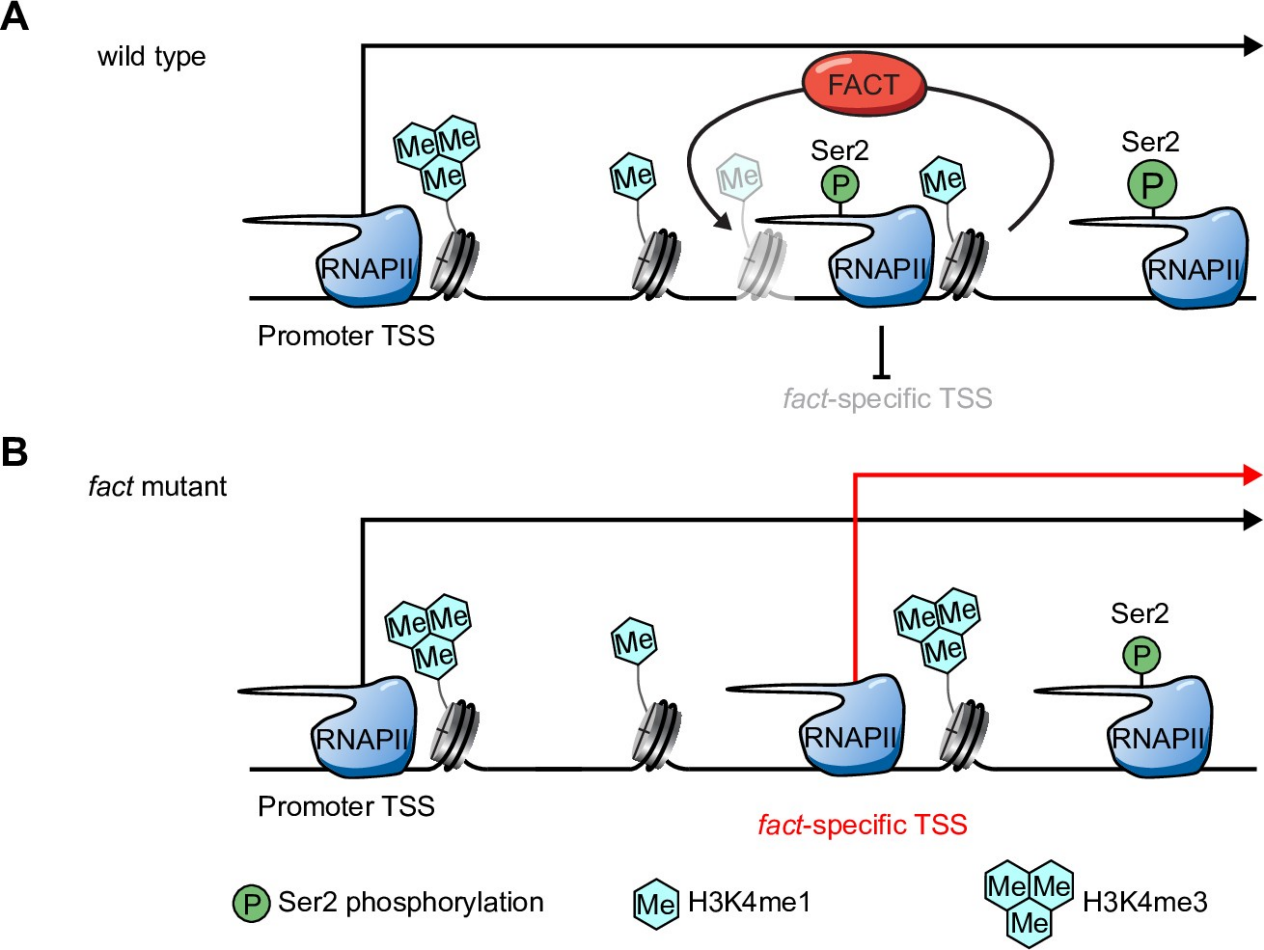
Cartoon summarizing FACT-dependent chromatin repression of intragenic TSSs in *Arabidopsis*. (A) In wild type *Arabidopsis*, RNAPII initiates transcription from canonical promoters of genes containing *fact*-specific TSS. Specific histone signatures such as H3K4me3 (blue tri-hexagon) are associated with TSSs at promoters, while RNAPII CTD Ser2 phosphorylation (green circle) and H3K4me1 (blue hexagon) are associated with RNAPII elongation zones. Repressed intragenic *fact*-specific TSSs are enriched for H3K4me1 in their repressed state. (B) *fact*-specific TSSs initiate transcription (red arrow) in *fact* mutants. Only a subset of intragenic sites marked with H3K4me1 represents *fact*-specific TSSs, indicated by a H3K4me1-marked nucleosome at a position without evidence for transcriptional initiation in *fact* mutants. Activation of *fact*-specific intragenic TSSs correlates with reduced H3K4me1 level and an increase of the H3K4me3 promoter signature.

All in all, our data support that FACT is required for the repression of intragenic TSSs in plants. Read-through transcription blurs transcript boundaries that may re-define gene promoters as intragenic, which reconciles the genetic requirement of FACT for promoter TSS repression by read-through transcription. Repression of promoter TSSs coincides with a loss of initiation-specific RNAPII hallmarks and a gain of elongation-specific signatures. Similarly, the FACT complex represses initiation of transcription from several thousand intragenic *fact*-specific TSSs. We could not fully resolve what molecularly distinguishes intragenic sites that function as *fact*-specific TSSs from surrounding locations, but *fact*-specific intragenic TSSs show relatively high levels of H3K4me1 in the repressed state. We condensed our results characterizing the chromatin dynamics accompanying the transition from FACT-repressed intragenic TSSs to active TSSs in a cartoon summarizing our findings (Fig 8). In conclusion, we uncover a co-transcriptional chromatin-based mechanism shaping gene regulation and transcript isoform diversity by regulating TSS selection in plants.

## Discussion

TSSs shape RNA isoform expression, but little is known about the mechanisms regulating TSS choice within transcription units. RNAPII transcription across gene promoters has the potential to re-define gene promoters as “intragenic” and repress them by mechanisms inhibiting initiation from within transcription units. We leveraged *Arabidopsis* T-DNA read-through mutants to identify a role of the conserved FACT histone chaperone complex in the repression of intragenic TSSs in a multicellular organism. Consistently, we identify a large number of intragenic TSSs repressed by FACT, particularly from exonic regions enriched for the chromatin signature H3K4me1.

Three activities of the FACT complex that may explain a key role in repressing intragenic TSSs across species are: 1.) stimulation of RNAPII elongation, 2.) histone re-assembly in the wake of RNAPII transcription to avoid gaps in nucleosome density, and 3.) recycling of old histones to maintain chromatin-based signals of POINS.

First, FACT stimulates RNAPII transcription of DNA templates packaged in nucleosome structures [30]. Structural analyses suggest that the FACT complex directly binds nucleosomes on several contacts of histone proteins, stabilizing otherwise energetically unfavorable nucleosome conformations that weaken nucleosome binding to DNA [78]. Stabilization of partly unfolded nucleosome intermediates facilitates RNAPII progression through nucleosome barriers. The ability to stabilize nucleosomes may distinguish FACT from other RNAPII elongation factors that did not score as hits in our assay, such as PAF-I or Elongator (S4 Fig). Defective FACT may result in “transcription stress” through stalled or arrested RNAPII molecules in transcription units that may trigger proteolytic degradation of stalled RNAPII [79]. Associated chromatin changes may facilitate the initiation of RNAPII transcription that could help to explain elevated TSSs in *fact* mutants [80, 81]. The relatively high H3K4me1 levels at *fact*-specific TSSs in wild type may indicate sites reliant on efficient RNAPII elongation. Consistently, we detect increased RNAPII ChIP-seq and GRO-seq signals at *fact*-specific TSSs compared to control regions in the same gene sets (Fig 6I and 6J). Consequently, defective elongation may contribute to preferential RNAPII initiation from within transcription units at these sites.

Second, FACT aids the re-assembly of nucleosomes from cellular histone pools in the wake of transcribing RNAPII to prevent gaps in nucleosome coverage [30, 82]. Consistently, reduced nucleosome density within transcription units has been reported in human and yeast *fact* mutants [40, 83]. Nucleosome Depleted Regions (NDRs) are associated with active promoter TSSs, and the establishment of intragenic NDRs may trigger the initiation of RNAPII transcription [28, 40, 84]. Relatively high MNase-seq signal at *fact*-specific TSSs compared to basal TSSs and control regions provides some evidence for nucleosomes blocking access to *fact*-specific TSSs (Fig 6A). FACT activity may be needed for TSS repression as nucleosome positioning at *fact*-specific TSSs may be sensitive to FACT histone re-assembly activity. Our locus-specific H3-qChIP analyses provide some support for this idea, as we detect a trend towards reduced H3 levels in *ssrp1-2* mutants at *fact*-specific TSSs. However, the reduction of H3 is statistically significant at only two out of four *fact*-specific TSSs (S9 Fig). Intragenic NDRs resulting from reduced FACT histone re-assembly activity may contribute to the increase of transcriptional initiation from *fact*-specific TSSs.

Third, the propensity of FACT to re-deposit histones back into their previous locations in the wake of RNAPII transcription represents an intuitive mechanism to maintain the co-transcriptional positional information provided by chromatin signatures [85]. The gradient of H3K4me at yeast genes from H3K4me3 at the beginning of genes towards H3K4me1 at the ends supports a role of differential methylation at H3K4 as a positional signal [18]. Old histones accumulate towards the H3K4me3-rich 5’ ends of yeast genes, so conceivably FACT may contribute to the co-transcriptional maintenance of this pattern [86]. Consistently, defective FACT disrupts POINS as is evidenced by the incorporation of the promoter-enriched histone variant H2A.Z within transcription units in yeast *fact* mutants [87]. Our analyses of H2A.Z ChIP-seq data found no evidence for high H2A.Z levels at *fact*-specific TSSs in wild type (S7 Fig). However, promoter TSSs chromatin signatures, such as H2A.Z, may accumulate at these sites in *fact* mutants. We find support for this idea in our qChIP analyses focused on other known active promoter chromatin signatures, such H3K4me3 (Fig 7), and to a lesser extent also H3K36me3 and H3K27ac (S11 and S12 Figs). Future studies will be required to dissect the contributions of defects in RNAPII elongation, nucleosome re-positioning, and POINS establishment in the up-regulation of intragenic TSSs observed in *fact* mutants.

In yeast, histone deacetylases associate with elongation-specific H3K36me3 and elongating RNAPII to repress the activity of intragenic TSSs [24, 25]. A reduction of histone acetylation in promoter regions in *qua1-1* and *rfd1-1* read-through mutants supports this observation in plants (Fig 3). Several histone deacetylases (HDACs) associate with RNAPII elongation complexes in *Arabidopsis* [42]. However, the plant HDAC complexes participating in the suppression of intragenic TSSs are yet to be identified. Our chromatin state analyses in *qua1-1* and *rfd1-1* support H3K36me2 as a chromatin signature of RNAPII elongation (Figs 3 and 6). Curiously, we find no evidence for a role of the *Arabidopsis* H3K36 methyltransferase *SDG8/ASHH2* in gene repression through the act of RNAPII transcription (S4 Fig). One of the 47 alternative SET-Domain Genes (SDGs) might contribute to the repression of intragenic TSSs [88]. Alternatively, since FACT-repressed intragenic TSSs are not specifically enriched for H3K36me2 compared to basal exonic TSSs and control regions in wild-type plants (Fig 6H, S6 Fig), TSS repression by the act of RNAPII elongation in plants may be less dependent on H3K36 methylation-based signals. Instead, our screen for chromatin signatures characterizing intragenic regions poised to function as TSSs in *fact* mutants identifies H3K4me1 as the strongest candidate histone variant or post-translational histone modifications enriched at these sites. Signals of H3K4me1 at *fact*-specific TSSs show an inverse relationship with increasing H3K4me3 levels when RNAPII initiates transcription from *fact*-specific intragenic TSSs (Fig 7). Perhaps, FACT is involved in recycling old H3K4me1-containing nucleosomes, since we detect reduced H3K4me1 at these sites in *fact* mutants. Newly incorporated nucleosomes might be more poised to accumulate H3K4me3 at these positions in *fact* mutants when transcription initiation is triggered from these sites (Fig 8). Alternatively, FACT-linked H3K4me3 demethylase- and/or H3K4me1 methyltransferase activities would be consistent with our results. However, the exact molecular mechanism of chromatin-based repression of intragenic TSSs in plants remains an area for future experimental focus.

The combination of H3K4me1, H3K27ac, and low levels of bidirectional transcription are classically associated with enhancer regions [89], however it is unclear if these features directly contribute to enhancer function [90]. Our analysis of H3K27ac ChIP-seq data showed reduced H3K27ac signals at *fact*-specific TSSs compared to basal TSSs (Fig 6B). We therefore disfavor the hypothesis that *fact*-specific intragenic TSSs are decorated with the combinatorial chromatin signatures characterizing enhancers. Intragenic H3K4me1 in *Arabidopsis* correlates with RNAPII elongation and counteracts H3K9me2-mediated gene repression [63]. While initiation of transcription from *fact*-specific TSSs can result in poly-adenylated RNA (Fig 7E and 7F), the overall expression is reduced compared to basal TSSs (Fig 5B and 5C). Selective RNA degradation shown for cryptic transcripts may offer a partial explanation [2]. Overall, some regions identified as *fact*-specific intragenic TSSs might bear similarity to mammalian “primed enhancers” that are poised for activation when new gene expression programs are implemented [91].

FACT activity is targeted by cancer therapeutics [92], yet the regulation of FACT activity in *Arabidopsis* is largely unexplored. We identified intragenic *fact*-specific TSSs using knock-down alleles, suggesting that relatively mild modulation of FACT activity elicits profound effects on intragenic initiation in *Arabidopsis*. *Arabidopsis spt16-1* and *ssrp1-2* mutants display similar phenotypic defects, indicating that regulation of intragenic TSSs may shape plant gene expression programs underlying environmental responses and development [44, 46]. A prime example may turn out to be plant light signaling that relies on alternative TSS choices [49], as we observed for the *AT4G15260* gene. Furthermore, recent examples of gene regulation by the act of interfering lncRNA transcription in yeast and human emphasize a key role for FACT [7, 37, 38]. While such examples remain to be characterized in plants, we demonstrate that the underlying mechanism of repressive RNAPII transcription is operational. Our study illustrates striking similarities between the repression of promoter TSSs by interfering read-through transcription and the repression of intragenic TSSs. These similarities can be reconciled by the repressive effects of RNAPII elongation on TSSs. While the underlying mechanism bears some overlap with classical studies in budding yeast, there appear to be important differences at the level of RNAPII elongation-associated chromatin signatures, highlighting functional differences between species.

Our study offers a platform to query the regulatory roles of intragenic TSSs in plants. We advance the molecular mechanism limiting intragenic TSSs by FACT. We map thousands of intragenic sites that initiate transcription when FACT function is compromised. Our data suggests that relatively high levels of H3K4me1 contribute to chromatin-based specification of these sites. Our insights into repressive RNAPII transcription promise to inform the characterization of gene regulation through the act of pervasive transcription throughout eukaryotic genomes.

## Methods

### Plant growth

All *Arabidopsis thaliana* lines used in this study are listed in the S5 Table. *Arabidopsis thaliana* and *Nicotiana benthamiana* plants were grown in greenhouses or climate chambers with a 16h light/8h dark cycle at 22°C for general growth and seed harvesting. For seedlings grown on plates, sterilized seeds were grown on 1/2 Murashige and Skoog (MS) medium containing 1% sucrose and supplemented with 1% Microagar.

### Growth of *rfd1-1* mutant plants

For analysis of the homozygous *rfd1-1* phenotype, seeds were sown in 96 well trays stratified for 2–3 days at 4°C. Plants for F2 analysis were grown in high light conditions (>100 μE). White seedlings were counted 10 days later. To propagate *rfd1-1* homozygotes, heterozygous *rfd1-1* seeds were sterilized and sown on MS plates with phosphinothricin selection, covered in foil, and stratified for 2 days at 4°C. Seeds were light induced for 6–8 h in a growth chamber with light strength of 80–100 μE. Plates were covered in foil for 3 days, the plates were unwrapped and grown in low light (<50 μE) for 3–4 weeks before transferring to soil. To isolate RNA, *rfd1-1* homozygote seeds and corresponding wild type controls were sterilized and sown on MS plates as described above and grown in low light for two weeks. In order to collect enough material for ChIP, heterozygous *rfd1-1* seeds were sterilized and sown on MS plates with phosphinothricin selection as described above and grown in low light for two weeks. Col-0 wild type controls were treated the same way, but without selection.

### Ruthenium red staining

Seeds were sown in 96-well plates containing 70 μl ddH_2_O. To synchronize germination, seeds were stratified at 4°C for 2–3 days. Germination of seeds was induced by light for 8–10 hours. The plates were wrapped in aluminium foil for 4 days. Etiolated seedlings were stained with 0.05% ruthenium red solution for 2 minutes. Seedlings were washed twice with ddH_2_O. Staining phenotype was recorded using a stereomicroscope.

### Cloning and plant transformation

Marker gene constructs were generated using pGWB vectors [93]. *TIp*_*RFD1*_ was amplified from *rfd1-1* genomic DNA using primers MLO414/422. *p35S* was amplified from *rfd1-1* genomic DNA using primers MLO538/MLO416. *TIp*_*RFD1*_ and *p35S* were inserted into the pENTR-D-Topo vector through topo reaction to generate entry vectors SMC358 (containing TIp_*RFD1*_) and SMC379 (containing *p35S*). Entry vectors were used in a LR reaction with pGWB533 (containing GUS) and pGWB540 (containing eYFP) to generate expression vectors SMC371 (*TIp*_*RFD1*_*-GUS*), SMC367 (*TIp*_*RFD1*_*-eYFP*), SMC377 (*p35S-GUS*) and SMC373 (*p35S-eYFP*). The expression vectors were transformed into *Agrobacterium tumefaciens* strain GV3850 by electroporation under 2.5kV, 400Ω resistance and 25uF capacitance. *Agrobacteria* harboring expression vectors were respectively co-infiltrated with the p19 suppressor of silencing into *Nicotiana benthamiana* and *Arabidopsis thaliana efr* mutant leaves [94]. GUS and eYFP signal was detected at 2 days after infiltration. Complementation constructs were generated using SMC330, a version of pEG302 [95] enabling hygromycin selection following plant transformation. SMC330 was generated by replacing the Bialaphos resistance gene with the Hygromycin resistance gene of pCambia1300. *TIp*_*QUA1*_:*QUA1* and TIp_*RFD1*_:*RFD1* were amplified from genomic wild type DNA using primers MLO727/728 and MLO414/442, respectively. The resulting PCR products were introduced into pENTR-D-Topo by topo cloning to generate entry vectors (SMC409 for *TIp*_*QUA1*_:*QUA1* and SMC356 for *TIp*_*RFD1*_:*RFD1*). The entry vectors were used in a LR reaction with SMC330 to generate expression vector SMC410 (containing *TIp*_*QUA1*_:*QUA1-FLAG* construct) and SMC380 (containing *TIp*_*RFD1*_:*RFD1-FLAG* construct). The complementation constructs were then transformed into *Agrobacterium tumefaciens* strain GV3101 (pMP90) by electroporation under 2.5kV, 400Ω resistance and 25μF capacitance. *Agrobacterium-*mediated transformation of *Arabidopsis* was performed as described in [96]. Homozygous *qua1-1* and heterozygous *rfd1-1 Arabidopsis* were used for complementation. Seeds from transformed *Arabidopsis* were screened for T-DNA integration by hygromycin resistance. Multiple independent single-locus insertions were identified by segregation analysis and tested for complementation and protein expression. Phenotypic complementation was tested using progeny of lines homozygous for *qua1-1* or *rfd-1*, and hemizygous for the complementation constructs (Fig 2D and 2F).

### β-glucuronidase (GUS) staining and fluorescence imaging

The GUS staining assay was performed as previously described [97]. X-Gluc (5-bromo-4-chloro-3-indolyl glucuronide) substrate was vacuum infiltrated into *A*. *thaliana* and *N*. *benthamiana* leaves. After staining, leaves were rinsed in 70% ethanol at room temperature until the chlorophyll was washed off. eYFP fluorescence was quantified using a Biorad imager Gel Doc.

### Western blotting

Equal amounts of plant material were harvested from plant tissue. Proteins were extracted in 2.5x extraction buffer (150 mM Tris-HCl pH 6.8; 5% SDS; 25% Glycerol; 0.025% Bromophenol blue; 0.1 mM DTT). Proteins were separated by SDS-PAGE using precast 4–15% Criterion TGX stain-free protein gels (Biorad) and transferred to PVDF membrane using a semi-dry Trans-blot Turbo transfer system (Biorad). Membranes were blocked (5% non-fat dried milk in PBS) for 1 hour at room temperature. Anti-FLAG (Sigma F3165) was added overnight at 4°C with rotation. Membranes were washed with PBS before incubation with the anti-mouse HRP-conjugated secondary antibody (Dako P0161) for 1 hour at room temperature. Membranes were washed in PBST. Chemiluminescent signals were detected using Super-Signal West Pico Chemiluminescent (Thermo Fisher Scientific) according to manufacturer’s instructions.

### Quantitative chromatin immunoprecipitation (qChIP)

qChIP experiments were performed essentially as described in [98], with minor modifications. For immunoprecipitations, Protein A magnetic beads (GenScript) and 2 μg of an antibody (Anti-Histone H3, ab1791; Anti-RNA polymerase II CTD YSPTSPS phosphor S2, ab5095; Anti-RNA polymerase II subunit B, AS11 1804; Anti-Histone H3 mono methyl K4, ab8895; Anti-Histone H3 tri methyl K4, ab8580; Anti-Histone H3 tri methyl K36, ab9050; Anti-Histone H3 di methyl K36, ab9049; Anti-Histone H3 pan-acetyl, ab47915; Anti-Histone H3 lysine 27 acetylation, ab4729) were added to solubilized chromatin. Quantitative analysis was performed on captured DNA by qPCR (Biorad). See S5 Table for oligonucleotide sequences. ChIP enrichments were calculated as the ratio of product of interest from IP sample to the corresponding input sample (%IP). For *qua1-1* and corresponding wild type (ecotype WS), %IP results were further normalized to %IP for an internal reference gene (*ACT2*) to account for different fixation conditions stemming from the *qua1-1* cell wall defect. Error bars represent standard error of the mean resulting from at least three independent replicates.

### RNA extraction and analyses

RNA was isolated from 14 day old seedlings using Plant RNeasy Mini-Kits as per manufacturer’s instructions (Qiagen). For RT–qPCR experiments, first strand complementary DNA synthesis was performed on Turbo DNase-treated (Ambion) RNA using oligo-dT primers and Superscript III (Invitrogen) as per manufacturer’s instructions. Negative controls lacking the reverse transcriptase enzyme (-RT) were performed alongside all RT–qPCR experiments. Quantitative analysis was performed by qPCR (Biorad). Data was normalized to an internal reference gene (*ACT2*). Levels in mutants represent relative expression compared to corresponding wild type. Northern analyses were performed as previously described with minor modifications [99]. Briefly, 5 micrograms of total RNA were separated by electrophoresis on agarose-formaldehyde-MOPS gels and transferred to a nylon transfer membrane by capillary blotting in 10x SSC overnight. RNA was crosslinked to the nylon membrane by UV irradiation. Membranes were probed with single stranded cDNA probes generated by incorporation of radioactive α-32P-dTTP. A Typhoon phosphoimager (GE Healthcare Life Sciences) was used for analysis. The general transcriptome sequence library (poly(A)-enriched) for RNA-seq of 2-week old *ssrp1-2* and wild type *Arabidopsis* seedlings were constructed using Illumina TruSeq Sample Prep Kit v2 following the manufacturer's protocol. Sequence library were measured on Agilent 2100 Bioanalyzer. The sequencing was performed on a HiSeq 4000 (Illumina) platform for paired-end 100 (PE100) run. 5’RACE experiments were performed using the SMARTer RACE 5'/3' Kit (Takara, Japan) according to manufacturer’s instructions. See S5 Table for oligonucleotide sequences.

### TSS-seq library construction

TSSs were mapped genome-wide in *Arabidopsis* using 5’-CAP-sequencing [60], with some minor changes as previously described [100]. Briefly, 5 micrograms of DNase-treated total RNA were treated with CIP (NEB) to remove all non-capped species in the sample. Next, 5’ caps were removed using Cap-Clip (CellScript) to permit ligation of single-stranded rP5_RND adapter to 5’-ends of previously capped species with T4 RNA ligase 1 (NEB). Poly(A)-enriched ligated RNAs were captured with oligo(dT) Dynabeads (Thermo Fisher Scientific) according to manufacturer’s instructions and fragmented in fragmentation buffer (50 mM Tris acetate pH 8.1, 100 mM KOAc, 30 mM MgOA) for 5 mins at 80°C. First-strand cDNA was generated using SuperScript III (Invitrogen) and random primers following manufacturer’s instructions. Second-strand cDNA was generated using Phusion high-fidelity polymerase (NEB) and the BioNotI-P5-PET oligo as per manufacturer’s instructions. Biotinylated PCR products were captured by streptavidin-coupled Dynabeads (Thermo Fisher Scientific), end repaired with End Repair Enzyme mix (NEB), A-tailed with Klenow fragment exo- (NEB), and ligated to barcoded Illumina compatible adapter using T4 DNA ligase (NEB). Libraries were amplified by PCR, size selected using AMPure XP beads (Beckman Coulter), pooled following quantification by Bioanalyzer (Agilent), and sequenced in single end mode on the following flowcell: NextSeq 500/550 High Output Kit v2 (75 cycles) (Illumina).

### Bioinformatic analysis

All custom code used in this study is available from https://github.com/Maxim-Ivanov/Nielsen_et_al_2018. Quality of raw TSS-seq data was consistently high as reported by the FastQC software (https://www.bioinformatics.babraham.ac.uk/projects/fastqc/). In brief, the TSS-seq data analysis pipeline was as follows: FASTQ files were subjected to quality and adapter trimming at 3' ends using Trim Galore v0.4.3 (—adapter "ATCTCGTATGCCG") (https://github.com/FelixKrueger/TrimGalore). UMI barcodes (8 nt) were trimmed from 5' ends and appended to FASTQ headers using UMI-Tools extract [101]. The adapter- and UMI-trimmed reads were aligned to TAIR10 genome assembly using STAR v2.5.2b (—outSAMmultNmax 1—alignEndsType Extend5pOfRead1) [102]. The output SAM files were sorted and converted to BAM using Samtools v1.3.1 [103]. Reads aligned to rRNA, tRNA, snRNA or snoRNA loci were filtered out using BEDTools v2.17.0 [104]. The resultant BAM files were filtered for reads with MAPQ≥10 using Samtools. Finally, BAM files were deduplicated using UMI-Tools dedup. The "clean" BAM files were converted to stranded Bedgraph files using BEDTools genomecov (-bg -5 -strand + for forward strand, -bg -5 strand—for reverse strand). Bedgraph files were compressed to BigWig format using kentUtils bedGraphToBigWig (https://github.com/ENCODE-DCC/kentUtils). For more details on the TSS-seq read alignment pipeline, see the 01-Alignment_of_5Cap-Seq_data.sh file in the code repository.

At the next step, TSSs were called from BigWig files using the CAGEfightR package v1.0.0 [105] which is available from Bioconductor (https://bioconductor.org/packages/release/bioc/html/CAGEfightR.html) and also from author's repository on Github (https://github.com/MalteThodberg/CAGEfightR). Only genomic positions supported by at least two 5' tags in at least two libraries from the same genotype were considered as TSS candidates. Adjacent TSSs separated by not more than 20 bp were merged together into TSS clusters. The TSS clusters were annotated by intersection with various genomic features which were extracted from the TxDb.Athaliana.BioMart.plantsmart28 package. The package contains annotations from ENSEMBL Plant version 28 which combines TAIR10 and Araport11. In particular, proximal upstream regions were defined as [(gene start)-500bp, (gene start)-100bp] and promoters as [(gene start)-100bp, (gene start)+100bp]. Called TSSs were annotated by genomic location as either genic ("promoter", "proximal", "fiveUTR", "threeUTR"), intragenic ("exon", "intron", "antisense"), or intergenic. In case of conflicting annotations, a single annotation was chosen according to the following hierarchy: intergenic < antisense < intron < exon < threeUTR < fiveUTR < proximal < promoter. The full TSS calling pipeline was detailed in the 02-Calling_TSS_with_CAGEfightR.R script. The statistical analysis of genomic distribution of the called TSS was described in the 03-Exploratory_analysis_of_exonic_TSS.R file in the code repository. The differential motif enrichment analysis was done using the DREME software [106].

To investigate the possible correlations between *fact*-specific exonic TSS and various histone modifications in *Arabidopsis*, we re-analyzed the available histone H2A, H2B, H3 and RNAPII ChIP-seq datasets [62–65] [73–76], as well as an MNase-seq dataset [66]. All accession numbers are available from the S6 Table. Two of these ChIP-seq datasets are paired-end [66] [75] and the rest are single-end. The pipelines for remapping paired-end and single-end ChIP-seq data were detailed in 04-Remapping_Paired-End_ChIP-Seq_and_MNase-Seq.sh and 05-Remapping_Single-End_ChIP-Seq.sh files, respectively. In brief, the alignment to the TAIR10 genome was done using STAR v2.5.2b. The BAM files were sorted and filtered for MAPQ≥10. To convert BAMs into Bedgraph files which correctly represent the source of ChIP-seq or MNase-seq signal, one has to infer the coordinates of original inserts. Otherwise, if read length was smaller than the average insert size, then the sequencing depth is expected to peak around the true source of ChIP-seq signal instead of coinciding with it. This operation is trivial for paired-end data, because the insert size for each pair of reads is directly available from the TLEN field of BAM files (see 04-Remapping_Paired-End_ChIP-Seq_and_MNase-Seq.sh). However, for single-end data the average insert size first has to be guessed from the data itself, and then each read has to be resized from its 3' end to half of the insert size. Therefore, we used single-end ChIP-seq BAM files as input for MACS2 software (-g 1.35e+08 -m 3,50—half-ext—bdg) [107] and continued with the output Bedgraph files (see 05-Remapping_Single-End_ChIP-Seq.sh).

Two of the single-end ChIP-seq datasets mentioned above were treated in a slightly different way: i) The raw data in Solexa and SCARF formats [67] were converted to FASTQ as detailed in the 06-Convert_Solexa_and_SCARF.sh file; ii) The color space data from ABI Solid platform [62] were aligned with Bowtie v1.2.2 (-C—best). Otherwise these special datasets were processed as described in 05-Remapping_Single-End_ChIP-Seq_and_MNase-Seq.sh.

In addition, to investigate the expression level of genes containing exonic TSS of interest, we converted the original tracks from an *Arabidopsis* GRO-seq study to Bedgraph format [77] (see the 07-Convert_GRO-Seq_data.sh).

Finally, all the ChIP-seq, GRO-seq and MNase-seq Bedgraph files were used as input for the custom boxplot and metagene plotting pipelines (see 08-Boxplot_and_metagene_pipeline.R in the code repository). The control intervals shown on boxplots and metagenes were produced by choosing random positions in exons of two gene sets: i) Genes with basal TSS (9221 genes); ii) Genes with *fact*-specific TSS (5604 genes). For GRO-seq plots, we removed control positions located less than 200 bp from gene ends, because plant GRO-seq is known to produce exaggerated signal at pA sites [77].

For RNA-seq data processing, standard Illumina adapters were trimmed from both R1 and R2 by Trim Galore v0.4.3 (—paired—Illumina). Then the read pairs were aligned to TAIR10 using the STAR aligner v2.5.2b in the local mode (—outSAMmultNmax 1—alignEndsType Local). The output SAM files were sorted, filtered for MAPQ≥10 and converted to BAM format using SAMtools v1.3.1. Finally, Bedgraph files for visualization in the IGV browser were generated from BAM files using BEDtools v2.17.0 (-bg -split).

## Supporting information

S1 Fig *RFD1* and *QUA1* promoters and T-DNA insertions. (A) The 117 bp *TIp*_*QUA1*_ promoter in *qua1-1* contains the *QUA1* TSS (as detected by TSS-seq in wild type) and upstream TATA element (bold and underlined). The predominant TSS peak is highlighted in blue. The start codon is highlighted in red. (B) Detailed annotation and sequence of functional elements from p35s in *qua1-1* T-DNA insertion. Schematic diagram is given, corresponding DNA sequence derived from Sanger sequencing of genomic DNA in matching color is given below. BAR (Bialaphos Resistance) annotates the ORF conferring resistance to the plant herbicide phosphinothricin. Arrows within sequence depicts TSS corresponding to TSS1 and TSS2 found in *qua1-1*/*ssrp1-2* (See Fig 4D). (C) The 307 bp *TIp*_*RFD1*_ promoter in *rfd1-1* contains the *RFD1* TSS (as detected by TSS-seq in wild type) and upstream TATA-like element (bold and underlined). The predominant TSS peak is highlighted in blue. The start codon is highlighted in red. (D) Detailed annotation and sequence of functional elements from p35s in *rfd1-1* T-DNA insertion. Schematic diagram is given, corresponding DNA sequence derived from Sanger sequencing of genomic DNA in matching color is given below. BAR (Bialaphos Resistance) annotates the ORF conferring resistance to the plant herbicide phosphinothricin. A tetrameric repeat of the 35S enhancer (35S Enh) sequence is located near the T-DNA right border (RB).
(TIF)

S2 Fig *TIp*_*RFD1*_ drives eYFP reporter gene expression in *Arabidopsis*. (A) Transient expression of *eYFP* reporter gene under the control of *TIp*_*RFD1*_ in *Arabidopsis efr* mutant leaves. *p35s-eYFP* and *p19* (lacking *eYFP reporter gene*) are shown as positive and negative controls respectively. (B) Quantification of eYFP signal in panel A using ImageJ based on three replicates of three infiltrated leaves per construct. A single asterisk denotes p<0.05 and two asterisks denote p<0.01 between samples by Student’s t-test.
(TIF)

S3 Fig Technical controls for qChIP analyses. (A) Schematic representation of the *QUA1* locus, including position of primer pairs for qChIP across *QUA1* gene in wild type (WS). (B) RNAPII Ser2P profile across *QUA1* in wild type. For statistical tests, a single asterisk denotes p<0.05 between samples by Student’s t-test. qChIP across *QUA1* in wild type for (C) H3K36me2/H3, (D) H3K36me3/H3, (E) H3ac/H3 and (F) H3K4me3/H3. (G) Histone H3 qChIP across *QUA1* in wild type (WS). and *qua1-1*. Note: For comparisons between wild type (WS) and *qua1-1*, qChIP values were normalized to reference gene *ACT2* in order to control for differential fixation conditions between samples (See methods for more details). (H) Schematic representation of the *RFD1* locus, including position of primer pairs for qChIP. (I) Histone H3 qChIP across *RFD1* in wild type (Col-0) and *rfd1-1*. Error bars represent standard error of means resulting from at least three independent replicates.
(TIF)

S4 Fig Suppression of *rfd1-1* and *qua1-1* by *fact* mutants, but not other transcription elongation factor mutants tested. (A) Segregation analysis of *rfd1-1* white cotyledon phenotype. Phenotypic segregation demonstrates that the *fact* mutant *spt16-1* suppresses the *rfd1-1* phenotype. Wild type (n = 161), *RFD1/rfd1-1* (n = 752), and *RFD1/rfd1-1; SPT16/spt16-1* (n = 1045). Dashed line indicates the expected ratio (25%) of seedlings with the white cotyledon phenotype in *RFD1/rfd1-1* progeny. Binomial test was used to determine that segregation for the white cotyledon phenotype of *RFD1/rfd1-1* and *RFD1/rfd1-1*; *SPT16/spt16-1* are significantly different from expected 25% (p = 0.00046 and p = 7.33e-21, respectively). As the *rfd1-1/rfd1-1* phenotype was not transmitted with full penetrance in our experimental conditions in *rfd1-1/RFD1* progeny, Fisher’s exact test was used to determine the statistical significance between the different F2 phenotypic segregation ratios of *RFD1/rfd1-1*, and *RFD1/rfd1-1*; *SPT16/spt16-1* (p = 0.00031). (B) Segregation analysis by ruthenium red staining. Dashed line indicates the expected ratio (25%) of progenies from a *QUA1/qua1-1* parent to be *qua1-1/qua1-1*, which is stained by ruthenium red. Based on the expected pattern of phenotypic segregation the *fact* mutant *spt16-1* suppresses the *qua1-1* phenotype, while the H3K36 methyltransferase mutant *sdg8-2*, the Elongator subunit mutant *elo3-6*, or the PAF-I subunit mutant *vip6-4* do not. Wild type (n = 97), *qua1-1/QUA1* (n = 456), *QUA1/qua1-1*; *SPT16/spt16-1* (n = 1008), *QUA1/qua1-1*; *SDG8/sdg8-2* (n = 479), *QUA1/qua1-1*; *ELO3*/*elo3-6* (n = 1198), and *QUA1/qua1-1*; *VIP6*/*vip6-4* (n = 395). Binomial testing was used to determine if the phenotypic segregation ratios are significantly lower than the expected 25%. We find statistical significant different segregation of *QUA1/qua1-1*; *SPT16/spt16-1* (p = 0.02), while the ratios of *QUA1/qua1-1*; *SDG8/sdg8-2* (p = 0.49), *QUA1/qua1-1*; *ELO3*/*elo3-6* (p = 0.08) and *QUA1/qua1-1*; *VIP6*/*vip6-4* (p = 0.45) show no statistically significant difference compared to the expected 25%. (C) The *QUA1* and *SSRP1* loci are linked on *Arabidopsis* chromosome 3. Genetic linkage prevents an accurate analysis of *qua1-1* suppression by *ssrp1-2* using segregating populations as in (B). *n*.*s*. denotes Not Significant, a single asterisk denotes p<0.05, two asterisks denote p<0.01 and three asterisks denote p<0.001 between samples/ratio by either Fisher’s exact test or binomial test as indicated.
(TIF)

S5 Fig Genome-wide TSS mapping in *Arabidopsis*. (A) TSS-seq read distribution across expressed *Arabidopsis* genes from 0.5 kb upstream of transcription start site (TSS) to transcription end site (TES) in wild type, *spt16-1*, and *ssrp1-2*. (B) Reproducibility of two TSS-seq replicates in wild type, *spt16-1*, and *ssrp1-2*. The scatterplots show the log2-transformed and CPM-normalized number of sequencing reads in each TSS cluster between the biological replicate samples. (C) The fraction of basal- and *fact*-specific TSS clusters which overlap reported CAGE peak summits. (D) Screenshot of different TSSs corresponding to alternative mRNA isoforms of the *AT4G08390* gene. The shorter isoforms utilize a second in-frame ATG to produce an N-terminally truncated protein that is differentially targeted within the cell [61]. (E) Distribution of *fact*-specific exonic TSS positions across exons revealing no positional bias.
(TIF)

S6 Fig H3K4me1 is enriched at FACT-repressed TSS positions. Metagene plots show the mean ChIP-seq, GRO-seq and MNase-seq values along 400 bp windows centered at the following positions: i) Promoter TSS (green); ii) Control exonic positions in genes with basal exonic TSS (purple); iii) Control exonic position in genes with *fact*-specific TSS (blue); iv) Basal exonic TSS (salmon); v) *fact*-specific exonic TSS (olive). Shaded area shows normal-based 95% confidence intervals for standard error of the mean. The following datasets were included: (A) MNase-seq; (B) H3K27ac; (C, D) H3K9ac and H3K36me3; (E, F, G) H3K4me3, H3K4me2 and H3K4me1; (H) H3K36me2; (I) RNAPII ChIP-seq; (J) GRO-seq.
(TIF)

S7 Fig Boxplots and metagene plots for histone H3, H2A, and H2B modifications. Boxplots show the median distribution of ChIP-seq signal within the same 20 bp windows as in Fig 6. Metagene plots show the mean ChIP-seq signal along the same 400 bp windows as in S6 Fig. The following datasets were used: (A, B, C) H3K4me3, H3K4me3 and H3K4me1; (D, E) H2A and H2A.Z; (F) H2Aub; (G) H2B; (H) H2Bub. Data was plotted for the following categories: i) Promoter TSS (green); ii) Control exonic positions in genes with basal exonic TSS; iii) Control exonic position in genes with *fact*-specific TSS; iv) Basal exonic TSS (salmon); v) *fact*-specific exonic TSS (olive).
(TIF)

S8 Fig Activation of cryptic intragenic TSSs accompanies a shift from H3K4me1 to H3K4me3. Screenshot of RNA-seq and TSS-seq data from wild type and *ssrp1-2* depicting novel intragenic transcripts emerging from *fact*-specific TSSs (red arrows) at the (A) *AT3G56210* and (B) *AT5G51200* genes. Screenshot of TSS-seq data from wild type and *ssrp1-2* depicting basal exonic TSS at the (C) *AT5G13630* and (D) *AT1G06680* genes. qChIP for H3K4me1 at canonical promoter and *fact*-specific TSS positions for (E) *AT3G56210*, (F) *AT5G51200*, and at basal exonic TSS positions for (G) *AT5G13630* and (H) *AT1G06680*. qChIP for H3K4me3 at canonical promoter and *fact*-specific TSS positions for (I) *AT3G56210*, (J) *AT5G51200*, and at basal exonic TSS positions for (K) *AT5G13630* and (L) *AT1G06680*. Error bars represent standard error of the mean resulting from at least three independent replicates. For statistical tests, a single asterisk denotes p<0.05 between samples by Student’s t-test.
(TIF)

S9 Fig Histone H3 levels at qChIP loci in wild-type and *ssrp1-2*. qChIP for total Histone H3 levels at canonical promoters and *fact*-specific promoters in wild-type and *ssrp1-2* at genes (A) *AT5G18500*, (B) *AT4G15260*, (C) *AT3G65210*, and (D) *AT5G51200*. qChIP for H3 levels at basal exonic TSSs found in genes *AT5G13630* (E) and *AT1G06680* (F). Error bars represent standard error of the mean resulting from at least three independent replicates. For statistical tests, a single asterisk denotes p<0.05 between samples by Student’s t-test. (See S8 Fig for primer pair positions)
(TIF)

S10 Fig H3K36me2 levels at qChIP loci in wild-type and *ssrp1-2*. qChIP for H3K36me2 levels at canonical promoters and *fact*-specific promoters in wild-type and *ssrp1-2* at genes (A) *AT5G18500*, (B) *AT4G15260*, (C) *AT3G65210*, and (D) *AT5G51200*. qChIP for H3K36me2 levels at basal exonic TSSs found in genes *AT5G13630* (E) and *AT1G06680* (F). Error bars represent standard error of the mean resulting from at least three independent replicates. For statistical tests, a single asterisk denotes p<0.05 between samples by Student’s t-test. (See S8 Fig for primer pair positions)
(TIF)

S11 Fig H3K36me3 levels at qChIP loci in wild-type and *ssrp1-2*. qChIP for H3K36me3 levels at canonical promoters and *fact*-specific promoters in wild-type and *ssrp1-2* at genes (A) *AT5G18500*, (B) *AT4G15260*, (C) *AT3G65210*, and (D) *AT5G51200*. qChIP for H3K36me3 levels at basal exonic TSSs found in genes *AT5G13630* (E) and *AT1G06680* (F). Error bars represent standard error of the mean resulting from at least three independent replicates. For statistical tests, a single asterisk denotes p<0.05 between samples by Student’s t-test. (See S8 Fig for primer pair positions).
(TIF)

S12 Fig H3K27ac levels at qChIP loci in wild-type and *ssrp1-2*. qChIP for H3K27ac levels at canonical promoters and *fact*-specific promoters in wild-type and *ssrp1-2* at genes (A) *AT5G18500*, (B) *AT4G15260*, (C) *AT3G65210*, and (D) *AT5G51200*. qChIP for H3K27ac levels at basal exonic TSSs found in genes *AT5G13630* (E) and *AT1G06680* (F). Error bars represent standard error of the mean resulting from at least three independent replicates. For statistical tests, a single asterisk denotes p<0.05 between samples by Student’s t-test. (See S8 Fig for primer pair positions).
(TIF)

S1 Table NGS quality metrics of TSS-seq. Table shows the number of reads obtained at every step of the TSS-seq data analysis pipeline. For detailed clarification of each step, see the 01-Alignment_of_5Cap-Seq_data.sh file in the https://github.com/Maxim-Ivanov/Nielsen_et_al_2018 code repository.
(XLSX)

S2 Table Intersections between TSS-seq tag clusters and the previously published coordinates of CAGE peaks. *Arabidopsis* CAGE peaks from [48] were analyzed for intersections with TSSs that were identified in our TSS-seq data. Rows show different annotation categories of TSSs. Cells contain the percentage values of TSSs in each annotation category which were found intersecting with the summits of CAGE peaks. Columns show the intersection statistics for different groups of TSSs (All vs Basal vs *fact*-specific).
(XLSX)

S3 Table Annotations of TSSs. This table shows count and percentages of Basal and *fact*-specific TSS clusters in different annotation categories. Data from this table was visually represented on Fig 5A.
(XLSX)

S4 Table Genomic coordinates of all TSSs. This table shows genomic coordinates of all TSSs which were identified in our TSS-seq data. The fields are as follows: i) "Chr", "Start", "End" and "Strand": strand-specific coordinated of each tag cluster which was called as a TSS; ii) "Score": the average number of TSS-seq tags per library in given TSS; ii) "Summit": coordinate of the base having the strongest signal within given TSS; iii) "Annotation": the most probable functional description of the surrounding genomic region ("intergenic", "proximal", "promoter", "fiveUTR", "intron", "exon", "threeUTR" or "antisense"); iv) "Category": either "*fact*-specific" (TSS was detected in *spt16-1* and/or *ssrp1-2* mutants but not in wild type plants) or "Basal" (TSS does not seem to be specific for the *fact* mutants); v) "Gene_ID" and "Gene_name": the nearest *Arabidopsis* gene which either overlaps the TSS or is located within 500 bp on the same strand ("intergenic" and "antisense" TSS were not annotated by geneID and gene name).
(XLSX)

S5 Table Resource table. This table contains all materials and other resources involved in this study.
(DOCX)

S6 Table Accession numbers for previously published data used here. This table contains accession numbers and PMIDs for all genomics datasets used in this study.
(XLSX)

S7 Table Source data table. Data used to generate the figures of this manuscript are provided as single excel file. Sheets are used to organize data for each figure.
(XLSX)
