# Supplementary figures and images for: Transcription-driven chromatin repression of Intragenic transcription start sites
Source: PLoS Genet. 2019 Feb 1;15(2):e1007969. doi: 10.1371/journal.pgen.1007969 (PMC6373976; doi:10.1371/journal.pgen.1007969)

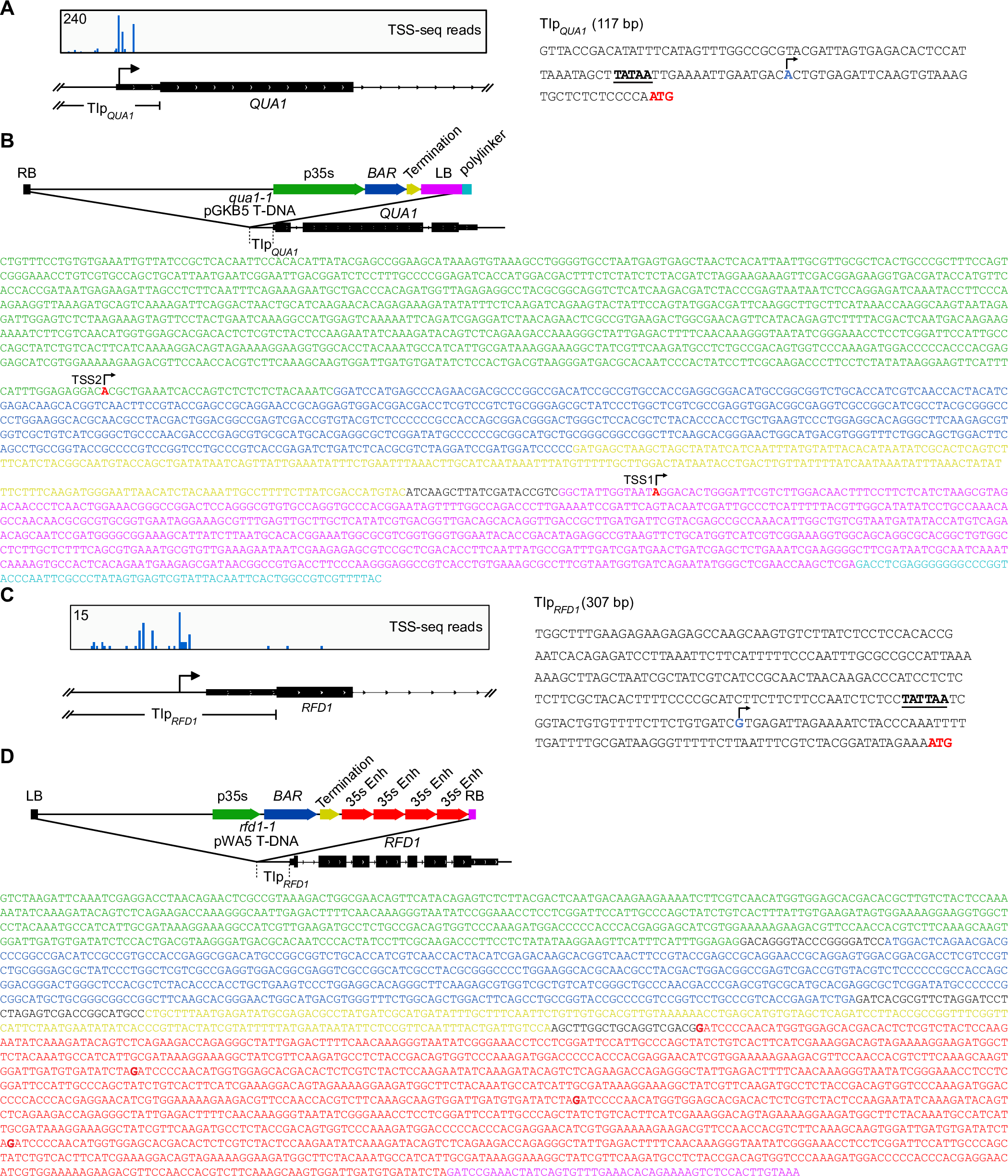

Supplement: S1 Fig — (A) The 117 bp TIpQUA1 promoter in qua1-1 contains the QUA1 TSS (as detected by TSS-seq in wild type) and upstream TATA element (bold and underlined). The predominant TSS peak is highlighted in blue. The start codon is highlighted in red. (B) Detailed annotation and sequence of functional elements from p35s in qua1-1 T-DNA insertion. Schematic diagram is given, corresponding DNA sequence derived from Sanger sequencing of genomic DNA in matching color is given below. BAR (Bialaphos Resistance) annotates the ORF conferring resistance to the plant herbicide phosphinothricin. Arrows within sequence depicts TSS corresponding to TSS1 and TSS2 found in qua1-1/ssrp1-2 (See Fig 4D). (C) The 307 bp TIpRFD1 promoter in rfd1-1 contains the RFD1 TSS (as detected by TSS-seq in wild type) and upstream TATA-like element (bold and underlined). The predominant TSS peak is highlighted in blue. The start codon is highlighted in red. (D) Detailed annotation and sequence of functional elements from p35s in rfd1-1 T-DNA insertion. Schematic diagram is given, corresponding DNA sequence derived from Sanger sequencing of genomic DNA in matching color is given below. BAR (Bialaphos Resistance) annotates the ORF conferring resistance to the plant herbicide phosphinothricin. A tetrameric repeat of the 35S enhancer (35S Enh) sequence is located near the T-DNA right border (RB). (TIF) [file pgen.1007969.s001.tif]

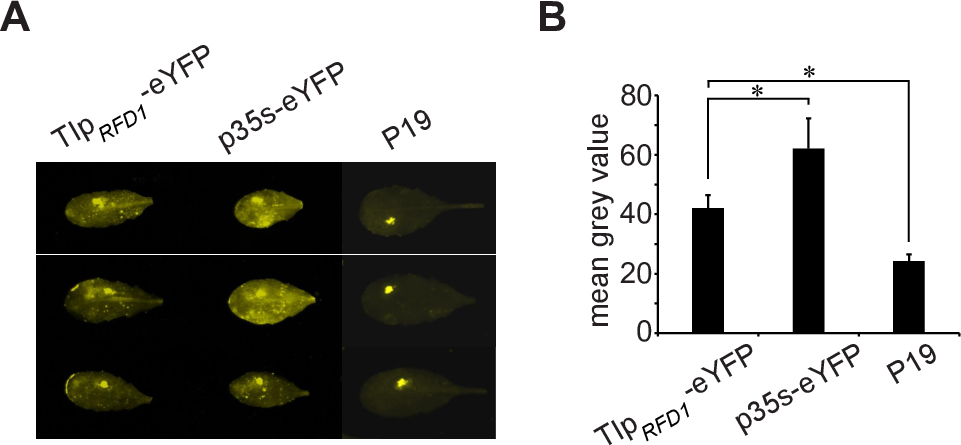

Supplement: S2 Fig — (A) Transient expression of eYFP reporter gene under the control of TIpRFD1 in Arabidopsis efr mutant leaves. p35s-eYFP and p19 (lacking eYFP reporter gene) are shown as positive and negative controls respectively. (B) Quantification of eYFP signal in panel A using ImageJ based on three replicates of three infiltrated leaves per construct. A single asterisk denotes p<0.05 and two asterisks denote p<0.01 between samples by Student’s t-test. (TIF) [file pgen.1007969.s002.tif]

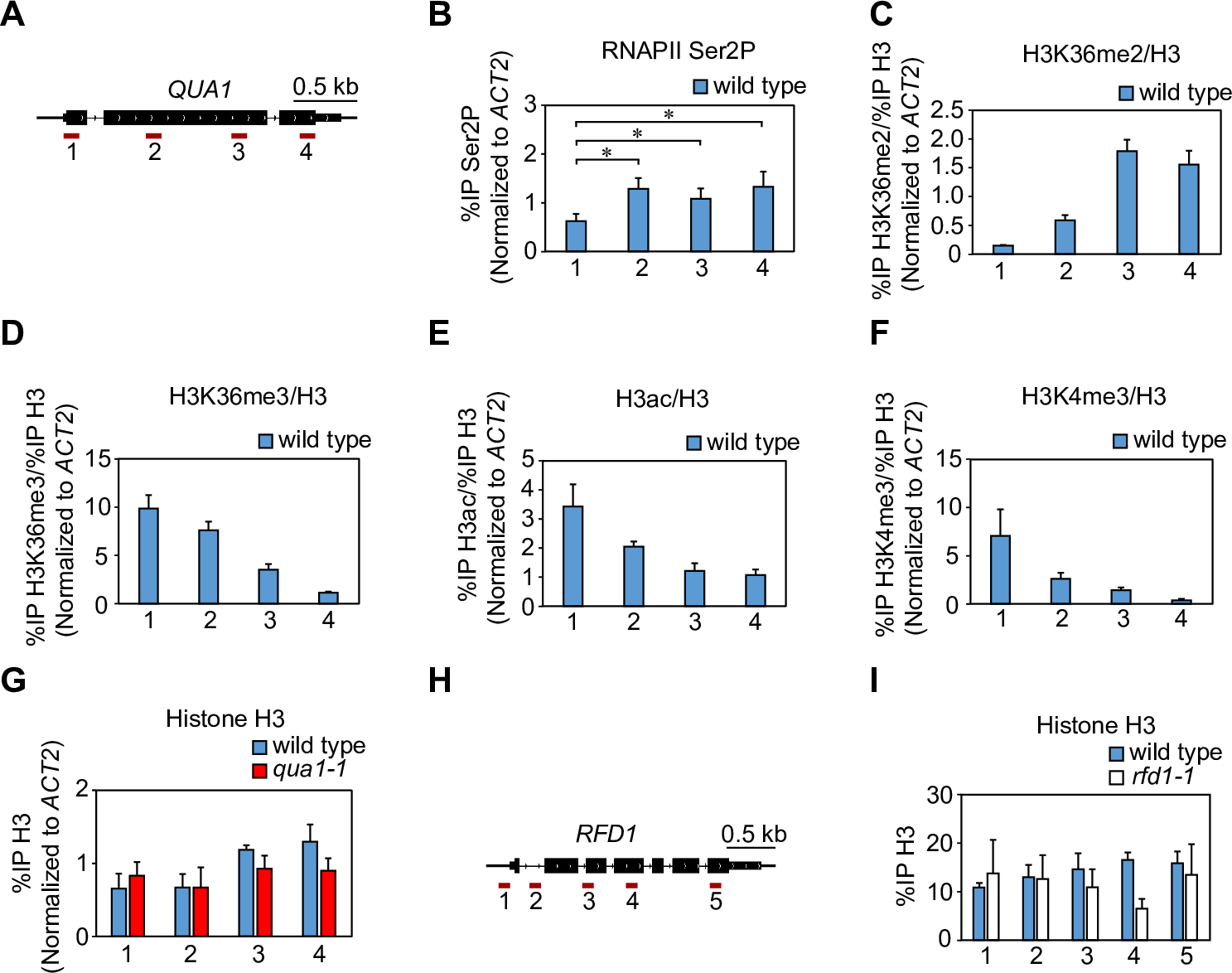

Supplement: S3 Fig — (A) Schematic representation of the QUA1 locus, including position of primer pairs for qChIP across QUA1 gene in wild type (WS). (B) RNAPII Ser2P profile across QUA1 in wild type. For statistical tests, a single asterisk denotes p<0.05 between samples by Student’s t-test. qChIP across QUA1 in wild type for (C) H3K36me2/H3, (D) H3K36me3/H3, (E) H3ac/H3 and (F) H3K4me3/H3. (G) Histone H3 qChIP across QUA1 in wild type (WS). and qua1-1. Note: For comparisons between wild type (WS) and qua1-1, qChIP values were normalized to reference gene ACT2 in order to control for differential fixation conditions between samples (See methods for more details). (H) Schematic representation of the RFD1 locus, including position of primer pairs for qChIP. (I) Histone H3 qChIP across RFD1 in wild type (Col-0) and rfd1-1. Error bars represent standard error of means resulting from at least three independent replicates. (TIF) [file pgen.1007969.s003.tif]

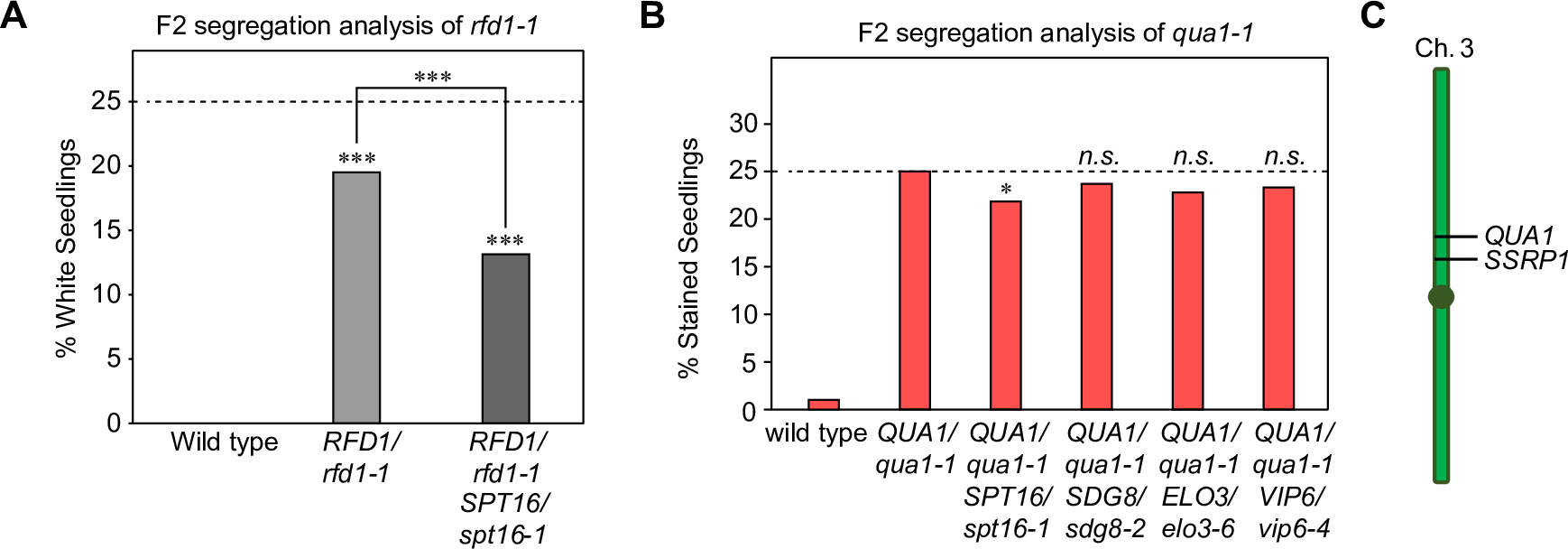

Supplement: S4 Fig — (A) Segregation analysis of rfd1-1 white cotyledon phenotype. Phenotypic segregation demonstrates that the fact mutant spt16-1 suppresses the rfd1-1 phenotype. Wild type (n = 161), RFD1/rfd1-1 (n = 752), and RFD1/rfd1-1; SPT16/spt16-1 (n = 1045). Dashed line indicates the expected ratio (25%) of seedlings with the white cotyledon phenotype in RFD1/rfd1-1 progeny. Binomial test was used to determine that segregation for the white cotyledon phenotype of RFD1/rfd1-1 and RFD1/rfd1-1; SPT16/spt16-1 are significantly different from expected 25% (p = 0.00046 and p = 7.33e-21, respectively). As the rfd1-1/rfd1-1 phenotype was not transmitted with full penetrance in our experimental conditions in rfd1-1/RFD1 progeny, Fisher’s exact test was used to determine the statistical significance between the different F2 phenotypic segregation ratios of RFD1/rfd1-1, and RFD1/rfd1-1; SPT16/spt16-1 (p = 0.00031). (B) Segregation analysis by ruthenium red staining. Dashed line indicates the expected ratio (25%) of progenies from a QUA1/qua1-1 parent to be qua1-1/qua1-1, which is stained by ruthenium red. Based on the expected pattern of phenotypic segregation the fact mutant spt16-1 suppresses the qua1-1 phenotype, while the H3K36 methyltransferase mutant sdg8-2, the Elongator subunit mutant elo3-6, or the PAF-I subunit mutant vip6-4 do not. Wild type (n = 97), qua1-1/QUA1 (n = 456), QUA1/qua1-1; SPT16/spt16-1 (n = 1008), QUA1/qua1-1; SDG8/sdg8-2 (n = 479), QUA1/qua1-1; ELO3/elo3-6 (n = 1198), and QUA1/qua1-1; VIP6/vip6-4 (n = 395). Binomial testing was used to determine if the phenotypic segregation ratios are significantly lower than the expected 25%. We find statistical significant different segregation of QUA1/qua1-1; SPT16/spt16-1 (p = 0.02), while the ratios of QUA1/qua1-1; SDG8/sdg8-2 (p = 0.49), QUA1/qua1-1; ELO3/elo3-6 (p = 0.08) and QUA1/qua1-1; VIP6/vip6-4 (p = 0.45) show no statistically significant difference compared to the expected 25%. (C) The QUA1 and SSRP1 loci are link [file pgen.1007969.s004.tif]

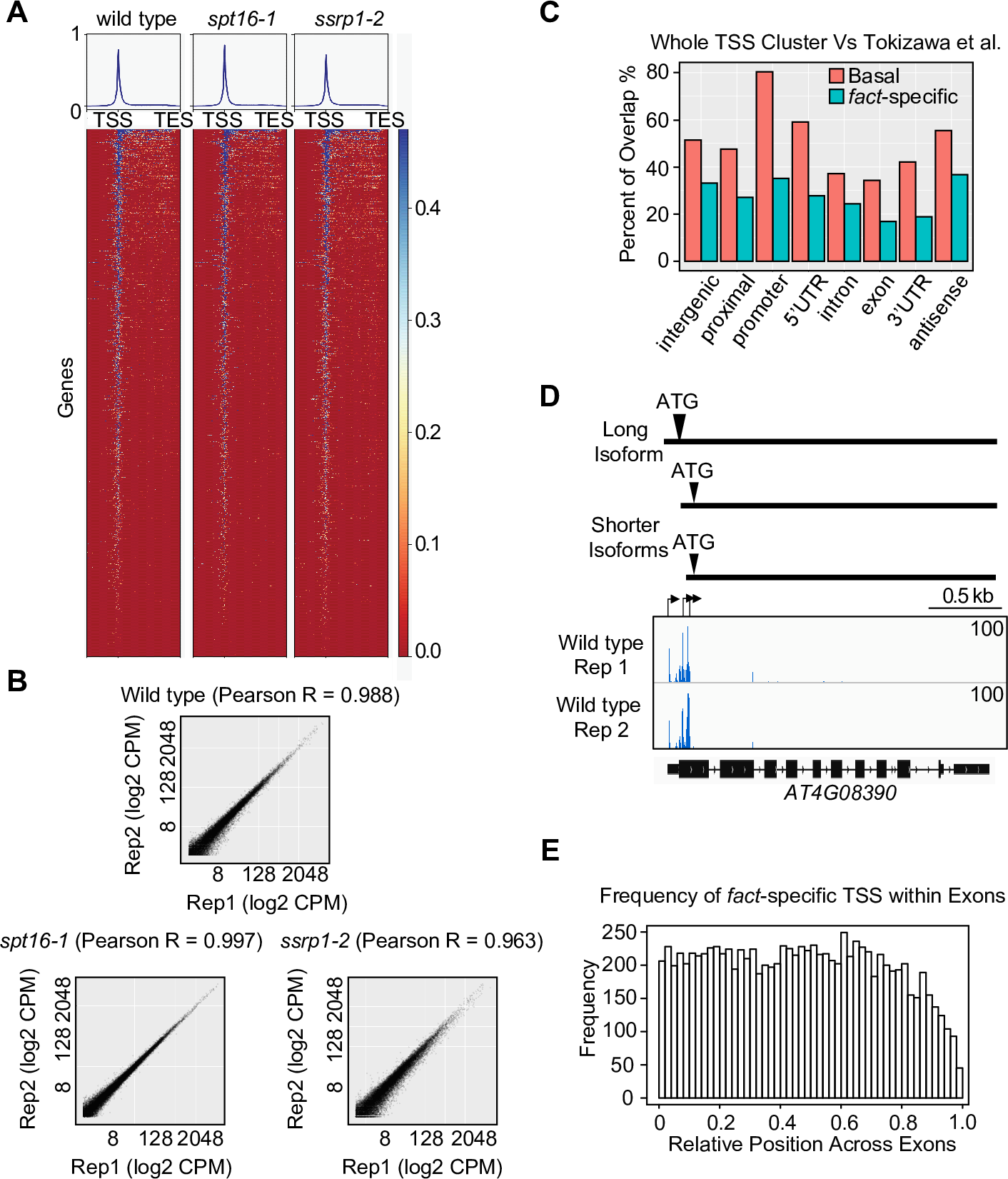

Supplement: S5 Fig — (A) TSS-seq read distribution across expressed Arabidopsis genes from 0.5 kb upstream of transcription start site (TSS) to transcription end site (TES) in wild type, spt16-1, and ssrp1-2. (B) Reproducibility of two TSS-seq replicates in wild type, spt16-1, and ssrp1-2. The scatterplots show the log2-transformed and CPM-normalized number of sequencing reads in each TSS cluster between the biological replicate samples. (C) The fraction of basal- and fact-specific TSS clusters which overlap reported CAGE peak summits. (D) Screenshot of different TSSs corresponding to alternative mRNA isoforms of the AT4G08390 gene. The shorter isoforms utilize a second in-frame ATG to produce an N-terminally truncated protein that is differentially targeted within the cell [61]. (E) Distribution of fact-specific exonic TSS positions across exons revealing no positional bias. (TIF) [file pgen.1007969.s005.tif]

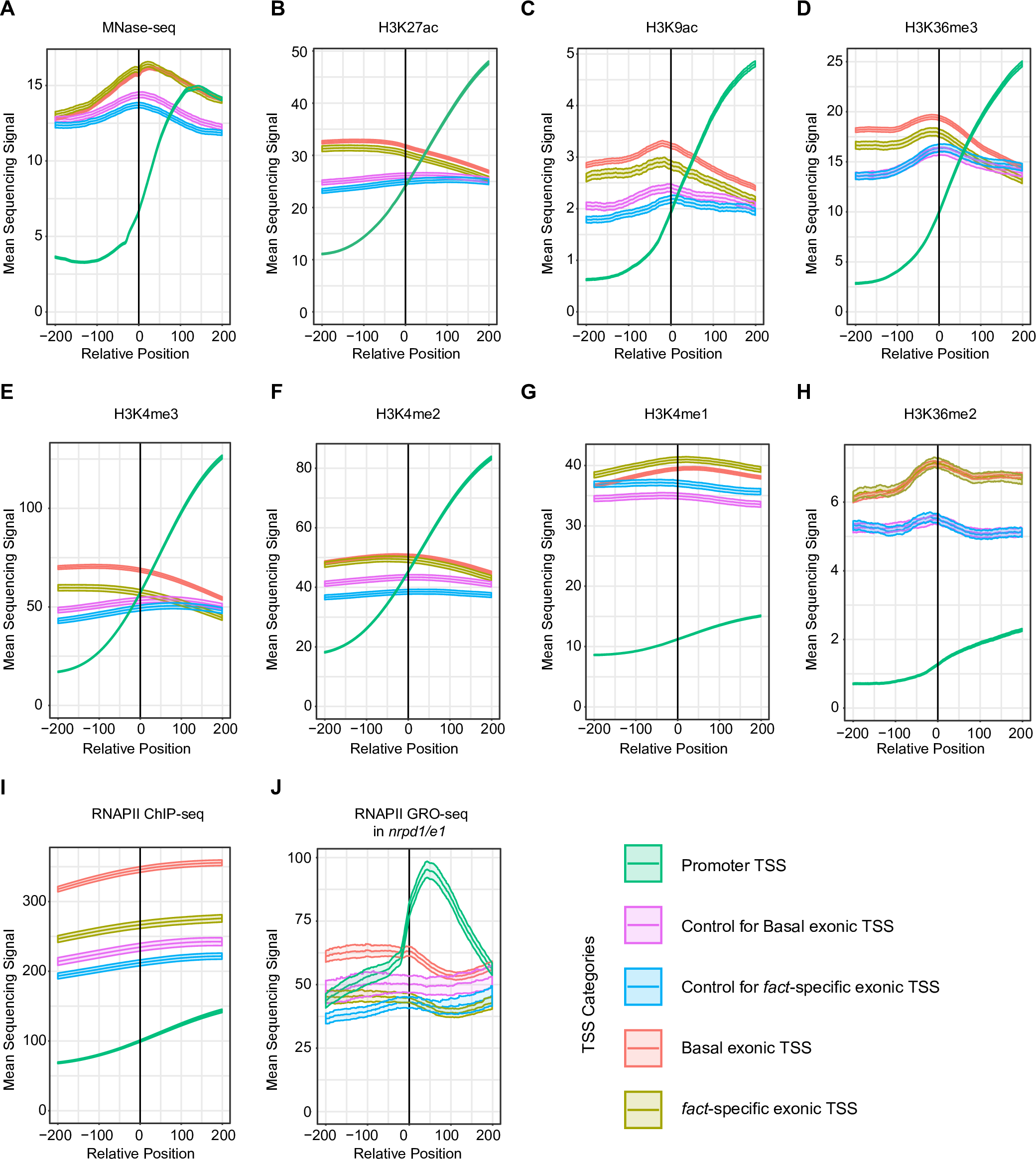

Supplement: S6 Fig — Metagene plots show the mean ChIP-seq, GRO-seq and MNase-seq values along 400 bp windows centered at the following positions: i) Promoter TSS (green); ii) Control exonic positions in genes with basal exonic TSS (purple); iii) Control exonic position in genes with fact-specific TSS (blue); iv) Basal exonic TSS (salmon); v) fact-specific exonic TSS (olive). Shaded area shows normal-based 95% confidence intervals for standard error of the mean. The following datasets were included: (A) MNase-seq; (B) H3K27ac; (C, D) H3K9ac and H3K36me3; (E, F, G) H3K4me3, H3K4me2 and H3K4me1; (H) H3K36me2; (I) RNAPII ChIP-seq; (J) GRO-seq. (TIF) [file pgen.1007969.s006.tif]

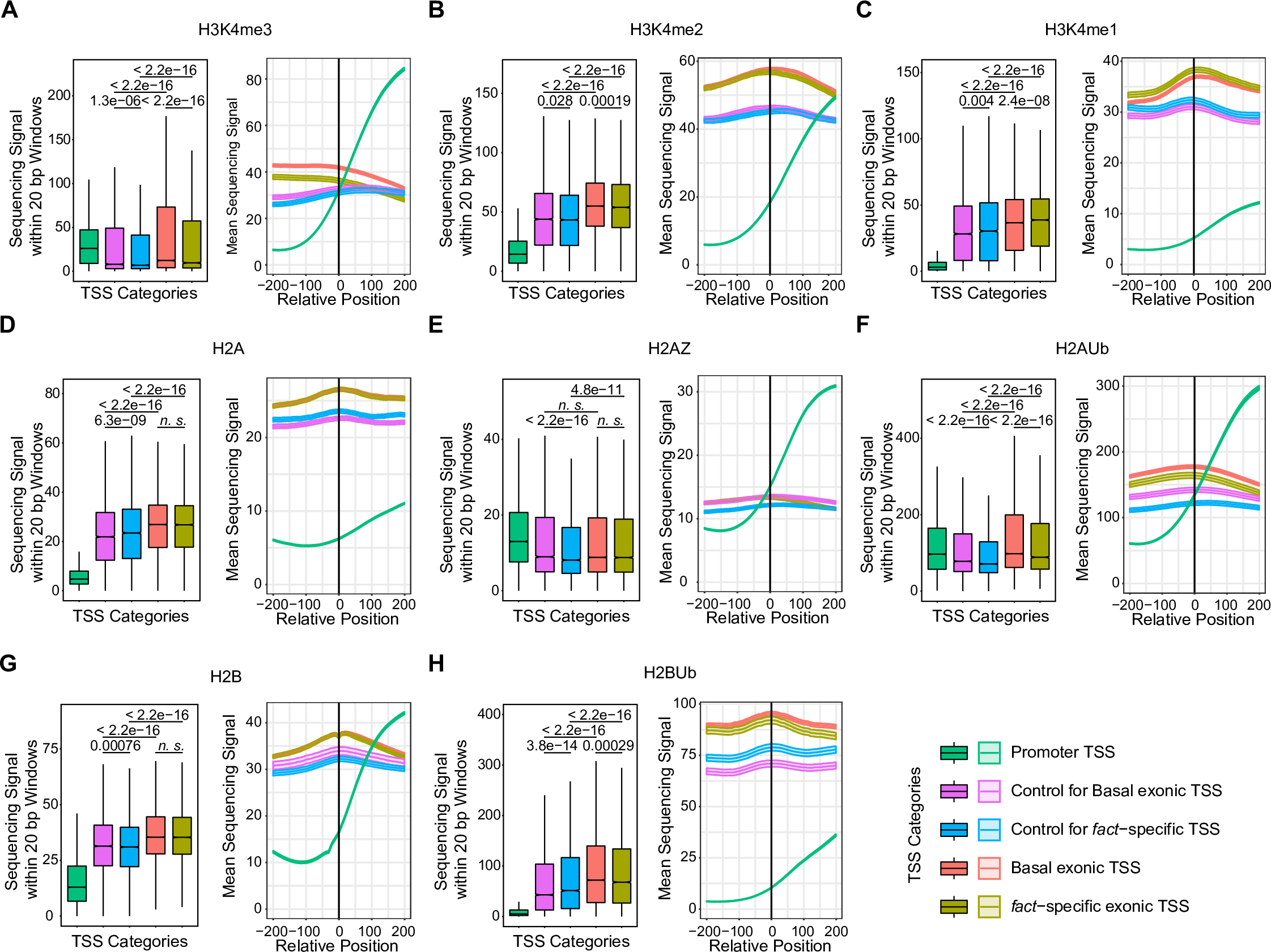

Supplement: S7 Fig — Boxplots show the median distribution of ChIP-seq signal within the same 20 bp windows as in Fig 6. Metagene plots show the mean ChIP-seq signal along the same 400 bp windows as in S6 Fig. The following datasets were used: (A, B, C) H3K4me3, H3K4me3 and H3K4me1; (D, E) H2A and H2A.Z; (F) H2Aub; (G) H2B; (H) H2Bub. Data was plotted for the following categories: i) Promoter TSS (green); ii) Control exonic positions in genes with basal exonic TSS; iii) Control exonic position in genes with fact-specific TSS; iv) Basal exonic TSS (salmon); v) fact-specific exonic TSS (olive). (TIF) [file pgen.1007969.s007.tif]

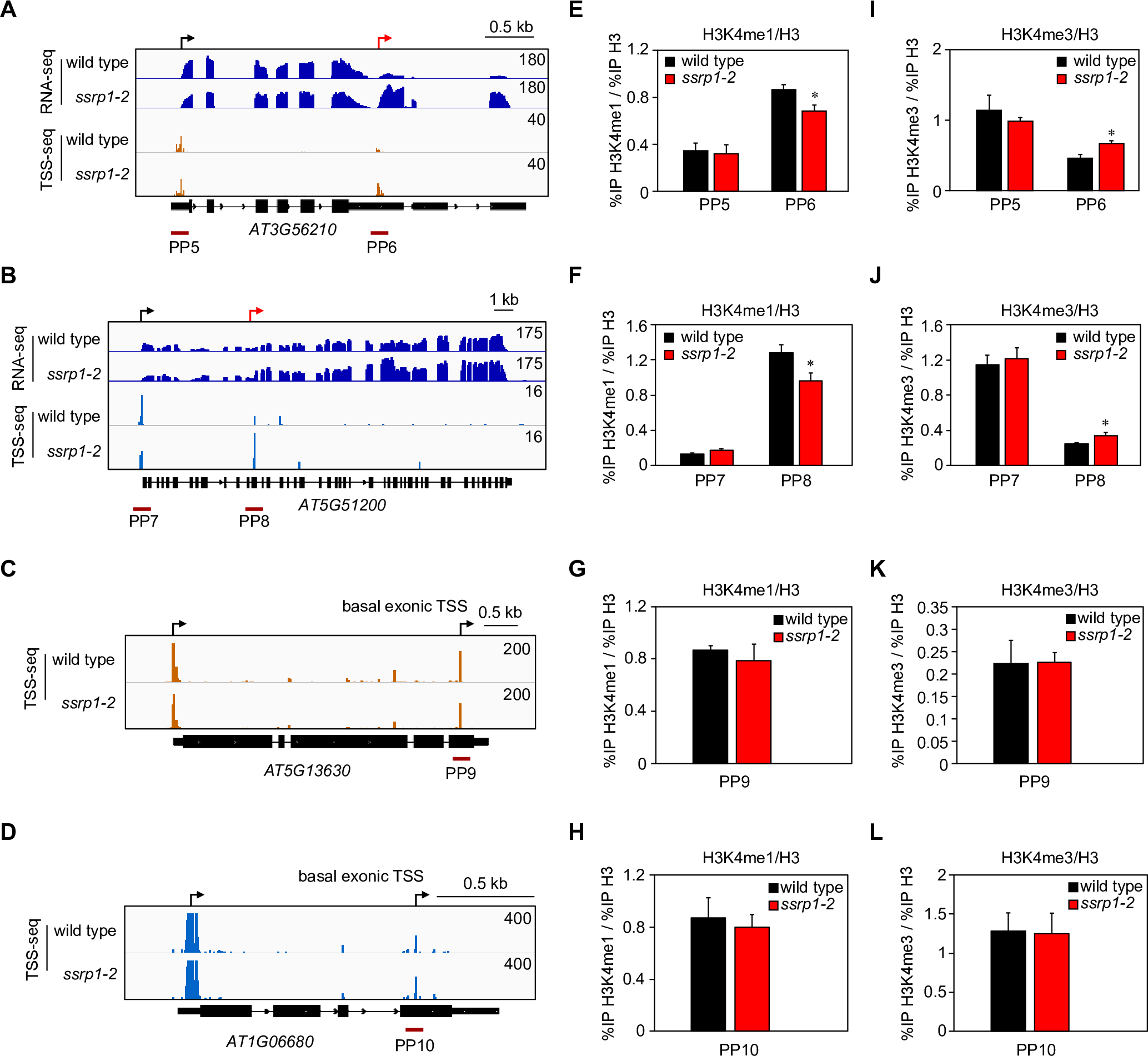

Supplement: S8 Fig — Screenshot of RNA-seq and TSS-seq data from wild type and ssrp1-2 depicting novel intragenic transcripts emerging from fact-specific TSSs (red arrows) at the (A) AT3G56210 and (B) AT5G51200 genes. Screenshot of TSS-seq data from wild type and ssrp1-2 depicting basal exonic TSS at the (C) AT5G13630 and (D) AT1G06680 genes. qChIP for H3K4me1 at canonical promoter and fact-specific TSS positions for (E) AT3G56210, (F) AT5G51200, and at basal exonic TSS positions for (G) AT5G13630 and (H) AT1G06680. qChIP for H3K4me3 at canonical promoter and fact-specific TSS positions for (I) AT3G56210, (J) AT5G51200, and at basal exonic TSS positions for (K) AT5G13630 and (L) AT1G06680. Error bars represent standard error of the mean resulting from at least three independent replicates. For statistical tests, a single asterisk denotes p<0.05 between samples by Student’s t-test. (TIF) [file pgen.1007969.s008.tif]

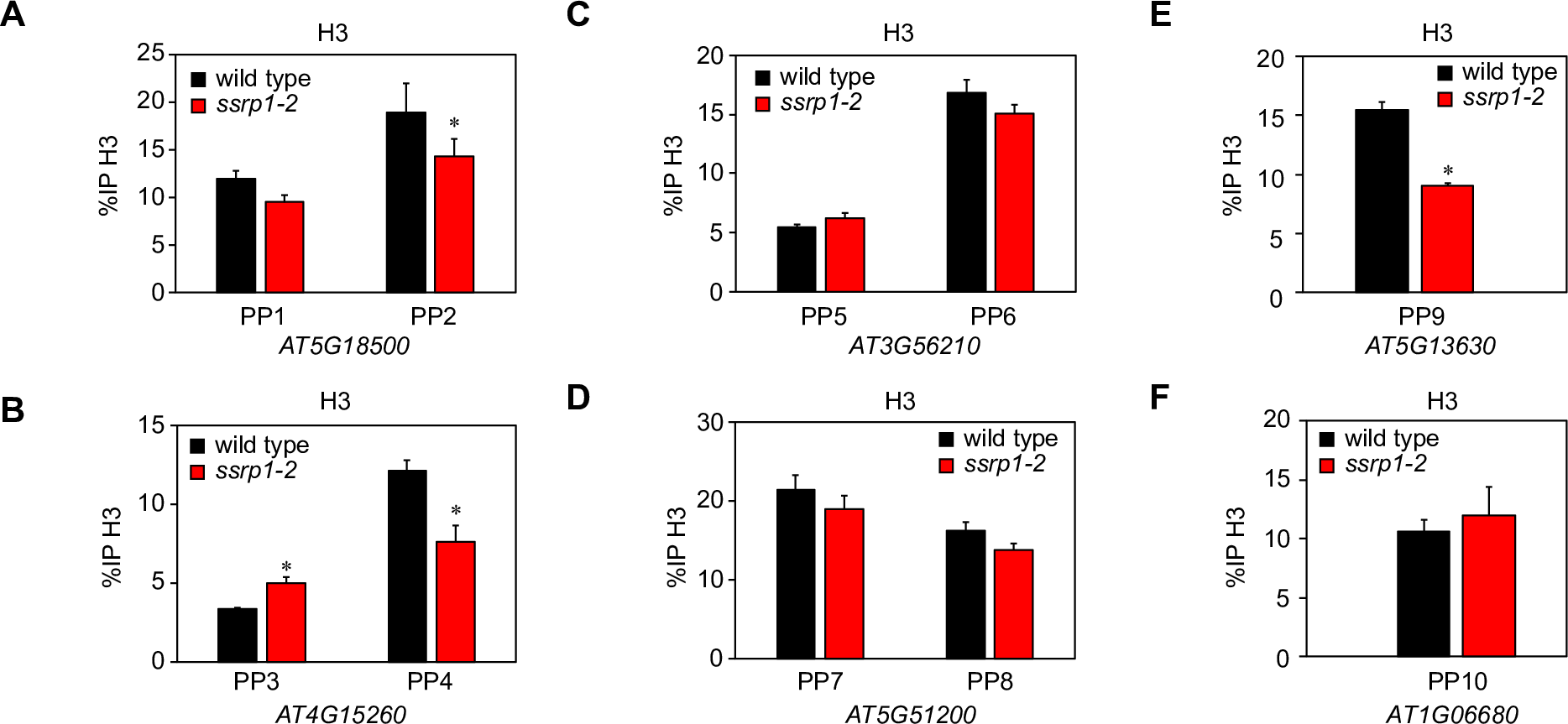

Supplement: S9 Fig — qChIP for total Histone H3 levels at canonical promoters and fact-specific promoters in wild-type and ssrp1-2 at genes (A) AT5G18500, (B) AT4G15260, (C) AT3G65210, and (D) AT5G51200. qChIP for H3 levels at basal exonic TSSs found in genes AT5G13630 (E) and AT1G06680 (F). Error bars represent standard error of the mean resulting from at least three independent replicates. For statistical tests, a single asterisk denotes p<0.05 between samples by Student’s t-test. (See S8 Fig for primer pair positions) (TIF) [file pgen.1007969.s009.tif]

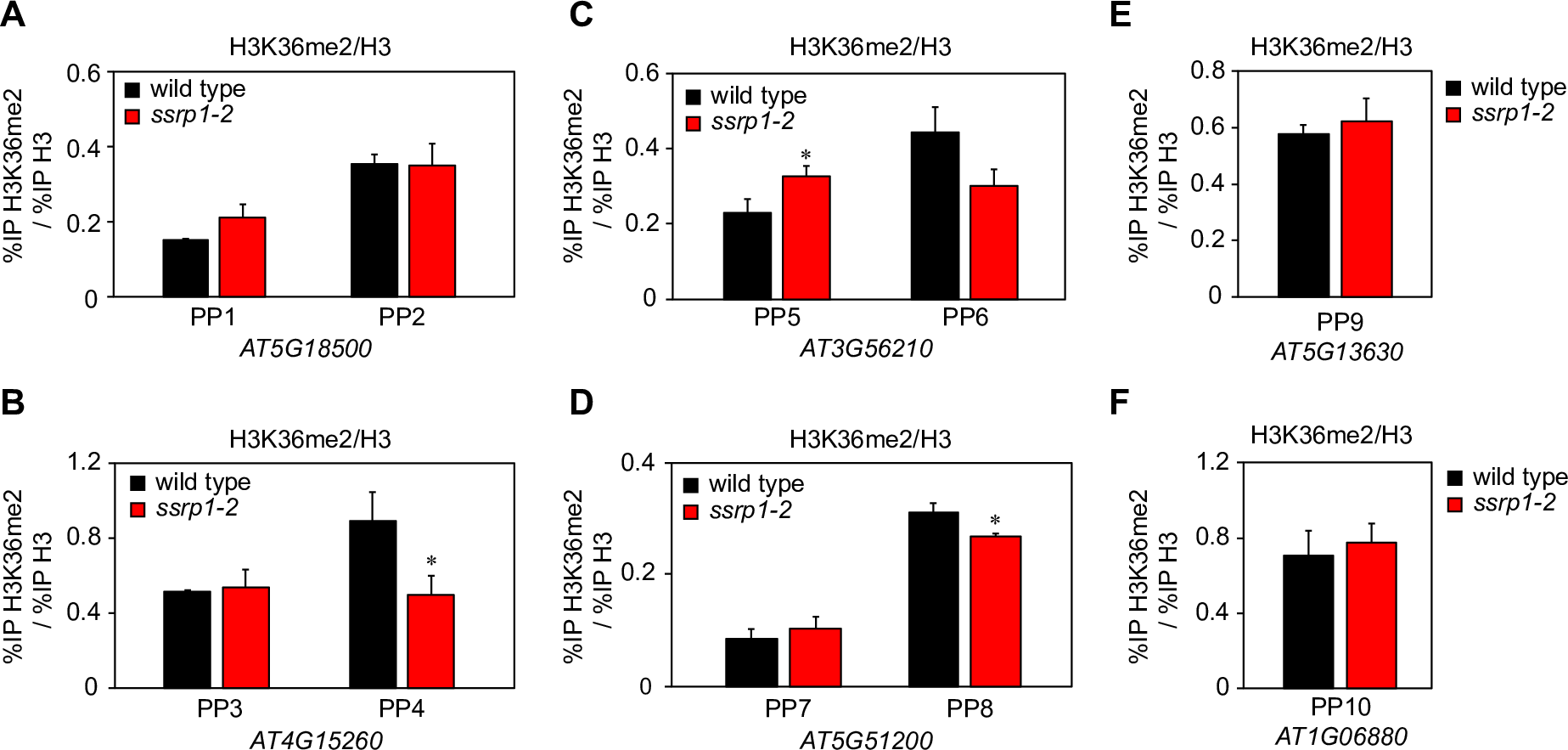

Supplement: S10 Fig — qChIP for H3K36me2 levels at canonical promoters and fact-specific promoters in wild-type and ssrp1-2 at genes (A) AT5G18500, (B) AT4G15260, (C) AT3G65210, and (D) AT5G51200. qChIP for H3K36me2 levels at basal exonic TSSs found in genes AT5G13630 (E) and AT1G06680 (F). Error bars represent standard error of the mean resulting from at least three independent replicates. For statistical tests, a single asterisk denotes p<0.05 between samples by Student’s t-test. (See S8 Fig for primer pair positions) (TIF) [file pgen.1007969.s010.tif]

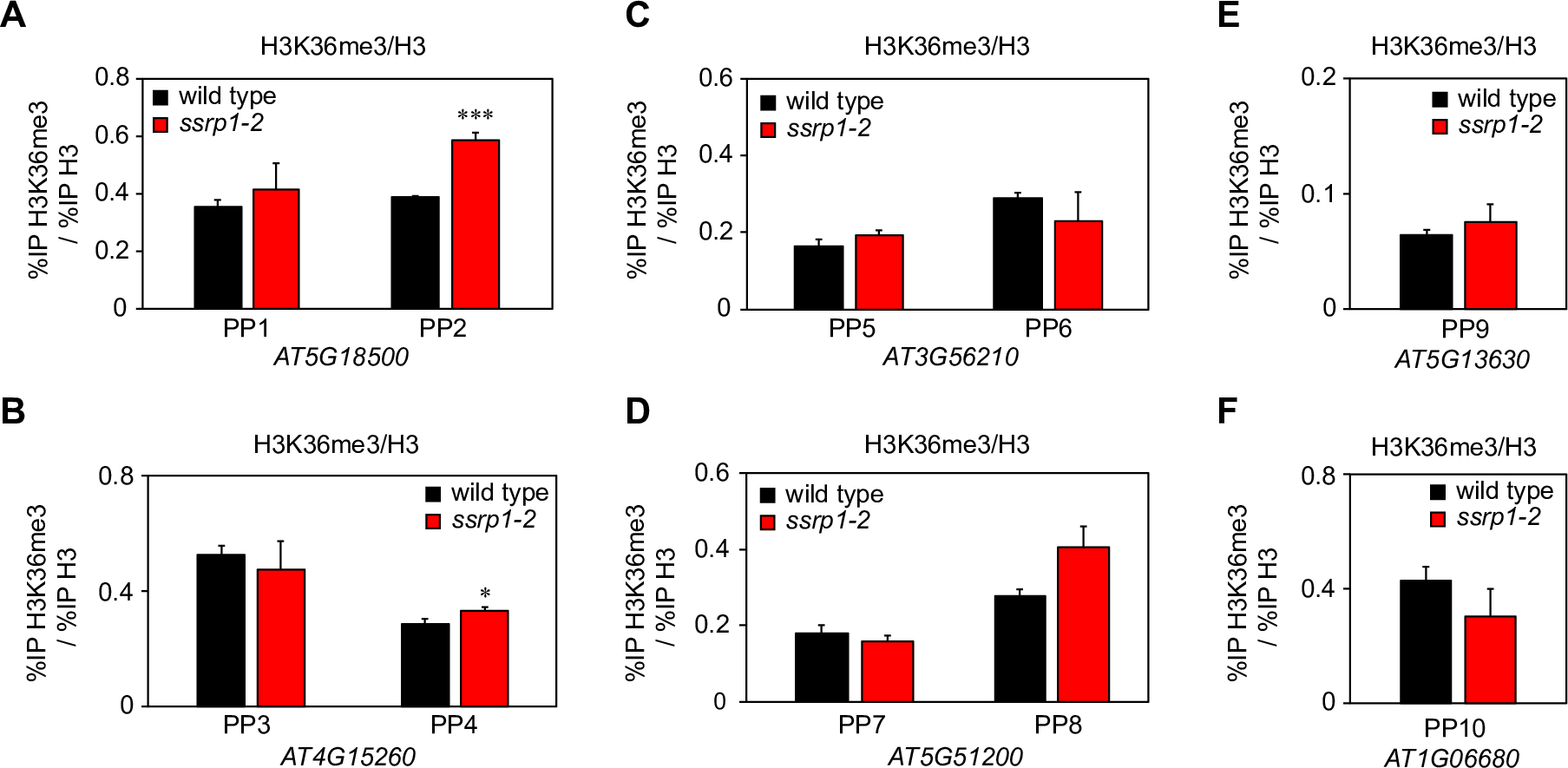

Supplement: S11 Fig — qChIP for H3K36me3 levels at canonical promoters and fact-specific promoters in wild-type and ssrp1-2 at genes (A) AT5G18500, (B) AT4G15260, (C) AT3G65210, and (D) AT5G51200. qChIP for H3K36me3 levels at basal exonic TSSs found in genes AT5G13630 (E) and AT1G06680 (F). Error bars represent standard error of the mean resulting from at least three independent replicates. For statistical tests, a single asterisk denotes p<0.05 between samples by Student’s t-test. (See S8 Fig for primer pair positions). (TIF) [file pgen.1007969.s011.tif]

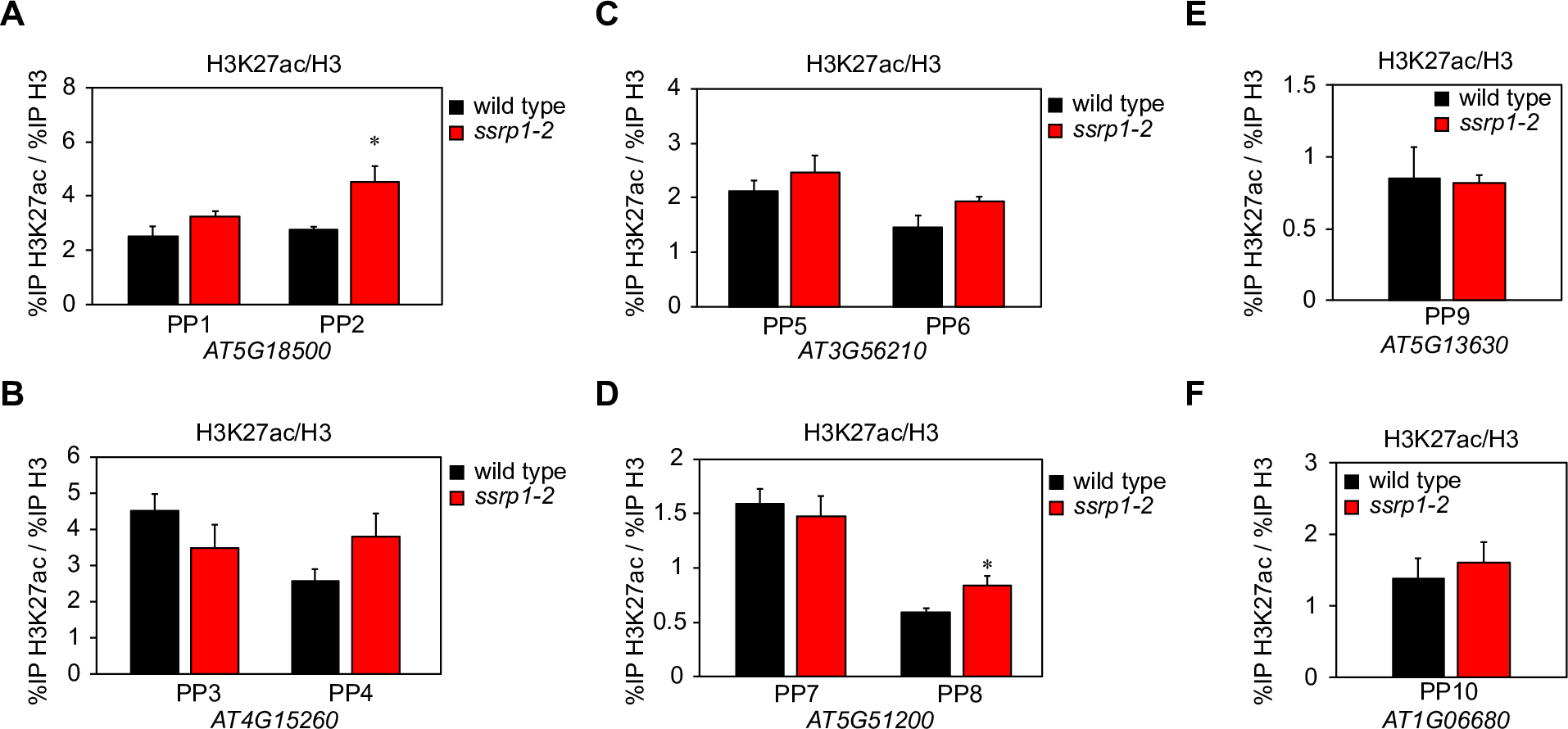

Supplement: S12 Fig — qChIP for H3K27ac levels at canonical promoters and fact-specific promoters in wild-type and ssrp1-2 at genes (A) AT5G18500, (B) AT4G15260, (C) AT3G65210, and (D) AT5G51200. qChIP for H3K27ac levels at basal exonic TSSs found in genes AT5G13630 (E) and AT1G06680 (F). Error bars represent standard error of the mean resulting from at least three independent replicates. For statistical tests, a single asterisk denotes p<0.05 between samples by Student’s t-test. (See S8 Fig for primer pair positions). (TIF) [file pgen.1007969.s012.tif]
