# Supplementary material for: Transcription-driven chromatin repression of Intragenic transcription start sites
Source: PLoS Genet. 2019 Feb 1;15(2):e1007969. doi: 10.1371/journal.pgen.1007969 (PMC6373976; doi:10.1371/journal.pgen.1007969)
Supplement: S5 Table — This table contains all materials and other resources involved in this study. (DOCX) [file pgen.1007969.s017.docx]

**Table 5:** Primers used in this study (Integrated DNA Technologies**^Ⓡ^**) and other resource information

| **Primers for Genotyping** | | |  |  |
| --- | --- | --- | --- | --- |
| **MLO #** | **Name** | **Sequence 5’-3’** | **Note** | |
| MLO20 | QUA1_F | CGTTCTGATATCCGAAATAAACATGTCTATTCA | Bouton et al, 2002 | |
| MLO21 | QUA1_BP | CTGATACCAGACGTTGCCCGCATAA | Bouton et al, 2002 | |
| MLO22 | QUA1_R | GTTGGATATGATCTGTTCGGAGAGAT | Bouton et al, 2002 | |
| MLO25 | pWA5-LB1 | TTTATAATAACGCTGCGGACATCTACATTTT | Hedtke et al, 2009 | |
| MLO539 | RFD1_F | TTTCTATATCCGTAGACGAAATTAAG |  | |
| MLO71 | RFD1_R | CGTCAAGGCAGAGACACATC |  | |
| MLO151 | SSRP1_F | ATGCTGCTGGTGGTGCTAA | Lolas et al, 2010 | |
| MLO152 | SSRP1_R | TGTTGCTTCTTAGCTATGGCCT | Lolas et al, 2010 | |
| MLO52 | SALK_BP | ATTTTGCCGATTTCGGAAC |  | |
| MLO35 | SPT16_P1 | CTATCTCTGCATTGCCTCTTAGC | Lolas et al, 2010 | |
| MLO36 | SPT16_P2 | TACTTGTCTAACGCAGCGAAATC | Lolas et al, 2010 | |
| MLO37 | SAIL_TDNA_P3 | GCCTTTTCAGAAATGGATAAATAGCCTTGCTTCC | Lolas et al, 2010 | |
| **Primers for cloning** | | | | |
| **MLO#** | **Name** | **Sequence 5’-3’** | **Note** | |
| MLO414 | Rfd1-gw_F | caccTGGCTTTGAAGAGAAGAGAG |  | |
| MLO416 | p35s core_R_1 | CTCTCCAAATGAAATGAACTTCC |  | |
| MLO422 | pRFD1_TIp_R_1 | TTTCTATATCCGTAGACG |  | |
| MLO442 | RFD1-Rw-last | GGACTCAGATTCAGACTCAATC |  | |
| MLO538 | New_p35s putative_F_3 | caccGAGTCTAAGATTCAAATCGAGGA |  | |
| MLO727 | TIp_in_qua1-1 | caccGTTACCGACATATTTCATAGTTTGGCCGC |  | |
| MLO728 | QUA1genR | GAGGCCAAAATTGCAAGCCTGAACA |  | |
| **Primers for Northern blotting** | | | | |
| **MLO#** | **Name** | **Sequence 5’-3’** | **Note** | |
| MLO895 | RFD1_1.exon_F | AGAGGATGGGTCTTGTTAGTTGC |  | |
| MLO415 | RFD1_lastexon_R | GGACTCAGATTCAGACTCAATCTTCTC |  | |
| MLO931 | QUA1_1.exon_F | ATGGCTAATCACCACCGACTTT |  | |
| MLO932 | QUA1_ lastexon_R | CCAAAATTGCAAGCCTGAACAAA |  | |
| **Primers for ChIP and RT-qPCR** | | | | |
| **MLO#** | **Name** | **Sequence 5’-3’** | **Note** | |
| MLO893 | RFD1_1F (Probe 1) | ACACCGAATCACAGAGATCCTTA | “promoter proximal primer pair” for ChIP | |
| MLO894 | RFD1_1R (Probe 1) | CAACTAACAAGACCCATCCTCT | “promoter proximal primer pair” for ChIP | |
| MLO895 | RFD1_1.1F (Probe 2) | AGAGGATGGGTCTTGTTAGTTGC |  | |
| MLO896 | RFD1_1.1R (Probe 2) | TCTAATCTCACGATCACAGAAGA |  | |
| MLO897 | RFD1_2F (Probe 3) | CATCGACACGAGAAGATATTCAA |  | |
| MLO898 | RFD1_2R (Probe 3) | CTAAGGTCACTGAAGCGCAATTA |  | |
| MLO899 | RFD1_3F (Probe 4) | CATTGAAGATATACGCCAAGGAA |  | |
| MLO900 | RFD1_3R (Probe 5) | ACCACAAGCTGCAACATTACC |  | |
| MLO903 | RFD1_5F (Probe 5) | TAGGACAATGAAGCTGATGACAA | Used for RT-qPCR | |
| MLO904 | RFD1_5R (Probe 5) | ACTCTCCCAACAATGGCTAATC | Used for RT-qPCR | |
| MLO931 | QUA1_1.1_F (Probe 1) | ATGGCTAATCACCACCGACTTT | “promoter proximal primer pair” for ChIP | |
| MLO908 | QUA1_1.1_R (Probe 1) | AGCGAAAGCTGTGAGTGTGA | “promoter proximal primer pair” for ChIP | |
| MLO909 | QUA1_3_F (Probe 2) | TTAACTAATGCTAAGAAGCAAGGTG |  | |
| MLO910 | QUA1_3_R (Probe 2) | ATCAGCCTCATAGCAAGACAATG |  | |
| MLO911 | QUA1_4_F (Probe 3) | GATGTGGTTGTGCAGAAGGAT |  | |
| MLO912 | QUA1_4_R (Probe 3) | AACAAGTCTCTACAGCTCCATTCA |  | |
| MLO913 | QUA1_5_F (Probe 3) | GGTACACTTCAACGGTAACATGA | Used for RT-qPCR | |
| MLO914 | QUA1_5_R (Probe 3) | CCTGAACAAACTCGAGGTCATAG | Used for RT-qPCR | |
| MLO455 | ACT2_F | CTTGCACCAAGCAGCATGAA |  | |
| MLO456 | ACT2_R | CCGATCCAGACACTGTACTTCCTT |  | |
| MLO1465 | AT5G18500 (PP1F) | GAACACAGCTACAAGGAGGGTAAA |  | |
| MLO1466 | AT5G18500 (PP1R) | CAGACAAAGAACCTAAGCAGTGAAA |  | |
| MLO1467 | AT5G18500 (PP2F) | AGTGATTGTTCTCTCCGCGATATTCG |  | |
| MLO1468 | AT5G18500 (PP2R) | TGGCGACTAACAGGGATTAGATTAG |  | |
| MLO1677 | AT4G15260 (PP3F) | CCTATGATTTGCATTAGACGAAACC |  | |
| MLO1678 | AT4G15260 (PP3R) | TAGTATAATTGGGCCGAGAACCTA |  | |
| MLO1679 | AT4G15260 (PP4F) | GAAGTGCCTTCCTCATATCCTTAGTTC |  | |
| MLO1680 | AT4G15260 (PP4R) | GCTCAAGCTCAGCAACTGTATTTACC |  | |
| MLO1575 | AT3G56210 (PP5F) | TCAGCTTAAGCATTGGAGAAGAGT |  | |
| MLO1576 | AT3G56210 (PP5R) | CTACCTGAGCTAAACAAGCCAGTC |  | |
| MLO1577 | AT3G56210 (PP6F) | TGGTATCCAATATACGTTGCTGTG |  | |
| MLO1578 | AT3G56210 (PP6R) | TACATTGGAGGAAGAAGCTGAGAG |  | |
| MLO1689 | AT5G51200 (PP7F) | CGAAAGATTTAGTGGCGATAGTACA |  | |
| MLO1690 | AT5G51200 (PP7R) | TTCTGAAGAGAGGGAAATGAGTTG |  | |
| MLO1691 | AT5G51200 (PP8F) | GGGTACATCATTTCGCAGTATAGG |  | |
| MLO1692 | AT5G51200 (PP8R) | TGAAGAGACTGCTTGAACTTCTCA |  | |
| MLO1693 | AT5G13630-PP9F | GAGTTGGCCTCCTCGTAGACCCA |  | |
| MLO1694 | AT5G13630-PP9R | AGTGGATATGAAGGAGTTCGTGAG |  | |
| MLO1695 | AT1G06680-PP10F | GTGTGAATCTGATGTTCTTACAGGGAG |  | |
| MLO1669 | AT1G06680- PP10R | GGTTTCCCACCAACTTCCTGAG |  | |
| **Primer for 5’RACE** | | | | |
| **MLO#** | **Name** | **Sequence 5’-3’** | **Note** | |
| MLO1473 | qua1_5RACE | GATTACGCCAAGCTTCTGCCACCGATTATGGCCGGAGATC |  | |
| **Primers for 5’-CAP-sequencing** | | | | |
| **MLO#** | **Name** | **Sequence 5’-3’** | **Note** | |
| N.A. | rP5_RND | CTTTCCCTACACGACGCTCTTCCGATrCrU**rNrNrNrNrNrNrNrN** | Ligated to 5’-end of decapped RNAs | |
| N.A. | BioNotI-P5-PET | ([Btn]TATAGCGGCCGCAATGATACGGCGACCACCGAGATCTA-CACTCTTTCCCTACACGACGCTCTTCCGATCT |  | |
| **Primers for pWA5 sequencing in *rfd1-1*** | | | | |
| **MLO#** | **Name** | **Sequence 5’-3’** | **Note** | |
| MLO772 | SynTI_seq_1F | TTCGCAAGACCCTTCCTCTA |  | |
| MLO773 | SynTI_seq_2F | GCCATCATTGCGATAAAGGA |  | |
| MLO774 | SynTI_seq_F2.2 | CTGTCACTTCATCGAAAGGA |  | |
| MLO775 | SynTI_seq_3R | CAGGAAACAGCTATGAC |  | |

**Resource Table:**

| RESOURCE | SOURCE | IDENTIFIER |
| --- | --- | --- |
| **Antibodies** | | |
| Monoclonal ANTI-FLAG® M2 antibody produced in mouse | Sigma-Aldrich | F3165; RRID: [AB_259529](http://antibodyregistry.org/AB_259529) |
| Anti-Histone H3 di methyl K36 | Abcam | Ab9049; RRID: AB_1280939 |
| Anti-Histone H3 tri methyl K4 | Abcam | Ab8580; RRID: AB_306649 |
| Anti-Histone H3 | Abcam | Ab1791; RRID :AB_302613 |
| Anti-RNA polymerase II subunit B | Agrisera | AS11 1804 |
| Anti-RNA polymerase II CTD phospho S2 | Abcam | Ab5095; RRID: AB_304749 |
| Anti-Histone H3 (acetyl K9+K14+K18+K23+K27) | Abcam | Ab47915; RRID: AB_873860 |
| Anti-Histone H3 tri methyl K36 | Abcam | Ab9050; RRID: AB_306966 |
| Anti-Histone H3 mono methyl K4 | Abcam | Ab8895; RRID:AB_306847 |
| Anti-Histone H3 (acetyl K27) | Abcam | Ab4729; RRID:AB_2118291 |
| **Bacterial Strains** | | |
| *E. coli* DH5α™ Competent Cells | Thermo Fisher | Cat no. 18265017 |
| *A. tumefaciens* GV3101(PMP90) | N/A | N/A |
| *A. tumefaciens* GV3850 | N/A | N/A |
| **Chemicals, Peptides, and Recombinant Proteins** | | |
| RNasin Plus RNase inhibitor | Promega | Cat. no. N2611 |
| Linear acrylamide | Thermo Fisher | Cat. no. AM9520 |
| Sodium acetate buffer solution | Sigma-Aldrich | Cat. no. S7899 |
| Calf intestinal alkaline phosphatase | NEB | Cat. no. M0290S |
| Cap-Clip acid pyrophosphatase | CellScript | Cat no. C-CC15011H |
| T4 RNA ligase 1 | NEB | Cat. no. M0204S |
| Klenow fragment | NEB | Cat. no. M0212S |
| 10× NEBuffer 2 | NEB | Cat. no. B7002S |
| dATP, 100 mM | NEB | Cat. no. N0440S |
| **Critical Commercial Assays** | | |
| QIAprep Spin Miniprep Kit (250) | QIAGEN | Cat no. 27106 |
| RNeasy Plant Mini Kit (50) | QIAGEN | Cat no. 74904 |
| Wizard^®^ SV Gel and PCR Clean-Up System | Promega | Cat no. A9182 |
| GoTaq^®^ qPCR Master Mix | Promega | Cat no. A6002 |
| SuperScript™ III Reverse Transcriptase | Thermo Fisher | Cat no. 18080093 |
| Dynabeads oligo (dT)25 mRNA isolation beads | Thermo Fisher | Cat no. 61002 |
| TURBO DNA-free kit | Thermo Fisher | Cat. no. AM1907 |
| Phusion high-fidelity (HF) PCR master mix | NEB | Cat. no. M0531S |
| Dynabeads M-280 streptavidin | Thermo Fisher | Cat. no. 11205D |
| NEBNext end repair module | NEB | Cat. no. E6050S |
| AMPURE XP Beads | Beckmann Coulter | Cat. no. A63880 |
| AMPURE XP Beads RNA | Beckmann Coulter | Cat. no. A66514 |
| Bioanalyzer DNA High Sensitivity kit | Agilent | Cat no. 5067-4626 |
| Qubit DNA high sensitivity | Thermo Fisher | Cat no. Q32854 |
| NextSeq® 500/550 High Output Kit v2 (75 cycles) | illumina | Cat no. FC-404-2005 |
| Protein A magnetic beads | GenScript | Cat. no. L00273 |
| SMARTer® RACE 5’/3’ Kit | Takara Bio USA, Inc. | Cat. no. 634859 |
| 4–15% Criterion™ TGX Stain-Free™ Protein Gel | BIO-RAD | Cat. no. 5678084 |
| TruSeq RNA Library Prep Kit v2 | illumina | Cat. No. RS-122-2001 |
| **Experimental Models: Organisms/Strains** | | |
| *Arabidopsis thaliana* wildtype Col-0 | N/A | N/A |
| *Arabidopsis thaliana* wildtype WS | N/A | N/A |
| *Arabidopsis thaliana* mutant *rfd1-1* | Hedtke and Grimm, 2009 | N/A |
| *Arabidopsis thaliana* mutant *qua1-1* | Bouton, 2002 | N/A |
| *Arabidopsis thaliana* mutant *sdg8-2* | NASC | SALK_026442 |
| *Arabidopsis thaliana* mutant *spt16-1* | NASC | SAIL_392_G06 |
| *Arabidopsis thaliana* mutant *ssrp1-2* | NASC | SALK_001283c |
| *Arabidopsis thaliana* mutant *elo3-6* | NASC | GK-555H06 |
| *Arabidopsis thaliana* mutant *vip6-4* | NASC | SALK_090130 |
| *Arabidopsis thaliana* mutant *efr-1* | Zipfel et al., 2006 | SALK_044334 |
| *Arabidopsis thaliana TIp_RFD1_:RFD1-Flag, rfd1-1* | this research | N/A |
| *Arabidopsis thaliana TIp_QUA1_:QUA1-Flag, qua1-1* | this research | N/A |
| *Arabidopsis thaliana* double mutant *qua1-1* *spt16-1* | this research | N/A |
| *Arabidopsis thaliana* double mutant *qua1-1* *ssrp1-2* | this research | N/A |
| *Arabidopsis thaliana* double mutant *qua1-1* *sdg8-2* | this research | N/A |
| *Arabidopsis thaliana* double mutant *qua1-1 elo3-6* | this research | N/A |
| *Arabidopsis thaliana* double mutant *qua1-1 vip6-4* | this research | N/A |
| *Nicotiana benthamiana* | N/A | N/A |
| **Software and Algorithms** | | |
| ImageJ | https://imagej.nih.gov/ij/ | N/A |
| Software for bioinformatic analysis: See Methods |  |  |
